# An unbiased approach to defining bona fide cancer neoepitopes that elicit immune-mediated cancer rejection

**DOI:** 10.1172/JCI142823

**Published:** 2021-02-01

**Authors:** Cory A. Brennick, Mariam M. George, Marmar M. Moussa, Adam T. Hagymasi, Sahar Al Seesi, Tatiana V. Shcheglova, Ryan P. Englander, Grant L.J. Keller, Jeremy L. Balsbaugh, Brian M. Baker, Andrea Schietinger, Ion I. Mandoiu, Pramod K. Srivastava

**Affiliations:** 1 Department of Immunology, and Carole and Ray Neag Comprehensive Cancer Center, University of Connecticut School of Medicine, Farmington, Connecticut, USA.; 2 Computer Science Department, Smith College, Northampton, Massachusetts, USA.; 3 Department of Chemistry and Biochemistry and Harper Cancer Research Institute, University of Notre Dame, Notre Dame, Indiana, USA.; 4 Proteomics and Metabolomics Facility, Center for Open Research Resources and Equipment, University of Connecticut, Storrs, Connecticut, USA.; 5 Immunology Program, Memorial Sloan Kettering Cancer Center, New York, New York, USA.; 6 Weill Cornell Medical College, Cornell University, New York, New York, USA.; 7 Department of Computer Science and Engineering, University of Connecticut, Storrs, Connecticut, USA.

**Keywords:** Immunology, Oncology, Antigen, Bioinformatics, Cancer immunotherapy

## Abstract

Identification of neoepitopes that are effective in cancer therapy is a major challenge in creating cancer vaccines. Here, using an entirely unbiased approach, we queried all possible neoepitopes in a mouse cancer model and asked which of those are effective in mediating tumor rejection and, independently, in eliciting a measurable CD8 response. This analysis uncovered a large trove of effective anticancer neoepitopes that have strikingly different properties from conventional epitopes and suggested an algorithm to predict them. It also revealed that our current methods of prediction discard the overwhelming majority of true anticancer neoepitopes. These results from a single mouse model were validated in another antigenically distinct mouse cancer model and are consistent with data reported in human studies. Structural modeling showed how the MHC I–presented neoepitopes had an altered conformation, higher stability, or increased exposure to T cell receptors as compared with the unmutated counterparts. T cells elicited by the active neoepitopes identified here demonstrated a stem-like early dysfunctional phenotype associated with effective responses against viruses and tumors of transgenic mice. These abundant anticancer neoepitopes, which have not been tested in human studies thus far, can be exploited for generation of personalized human cancer vaccines.

## Introduction

Mutational cancer neoepitopes are the only truly tumor-specific antigens. They are therefore the best candidates for cancer vaccines. However, only a very small proportion of all potential neoepitopes in a tumor are true tumor rejection–mediating neoepitopes (TRMNs) and their identification is a major challenge. The current methods for prediction of neoepitopes are based primarily on our understanding of MHC-peptide interactions (1). These rules have been derived from extensive study of viral epitopes and have stood the test of time. However, there is now increasing evidence from human and murine studies that these rules may not apply as neatly to the definition of TRMNs. The clinical trials using neoepitopes (2, 3) have consistently shown that “prominent CD4^+^ T cell responses against immunizing neoantigens were detected, despite the use of [MHC] class I binding prediction algorithms” (3). Ghorani et al. (4) and Rech et al. (5) examined mutational and clinical outcome data from several thousand patients and, remarkably, concluded that the presence of high-affinity MHC-binding neoepitopes in tumors showed no correlation with overall survival, progression-free survival, or T cell infiltration in tumors (4, 5). Previously, Duan et al. (6) had similarly reported lack of antitumor activity in high-affinity MHC I–binding neoepitopes in mouse models. Nelson and colleagues have reported a complete absence of antitumor activity among all the high-affinity MHC I–binding neoepitopes of a murine ovarian cancer (7).

In order to reconcile these conflicting results, we have queried all possible (nearly 300) neoepitopes using an entirely unbiased approach and asked which of those are effective in mediating tumor rejection and, independently, in eliciting CD8^+^ T cell response. These analyses revealed unexpected insights into the nature of TRMNs and the rules that may be used to predict them. They showed that our current methods of prediction discard the majority of true anticancer neoepitopes, and that the true TRMNs have strikingly different properties from epitopes of viral antigens. The identification of a relatively large number of true TRMNs and true non-TRMNs in an unbiased analysis also allows for creation of a preliminary algorithm for prediction of true TRMNs from in silico exome-sequencing data.

## Results

### Identification of single-nucleotide variants and TRMNs in MC38-FABF tumor.

**Figure 1:**
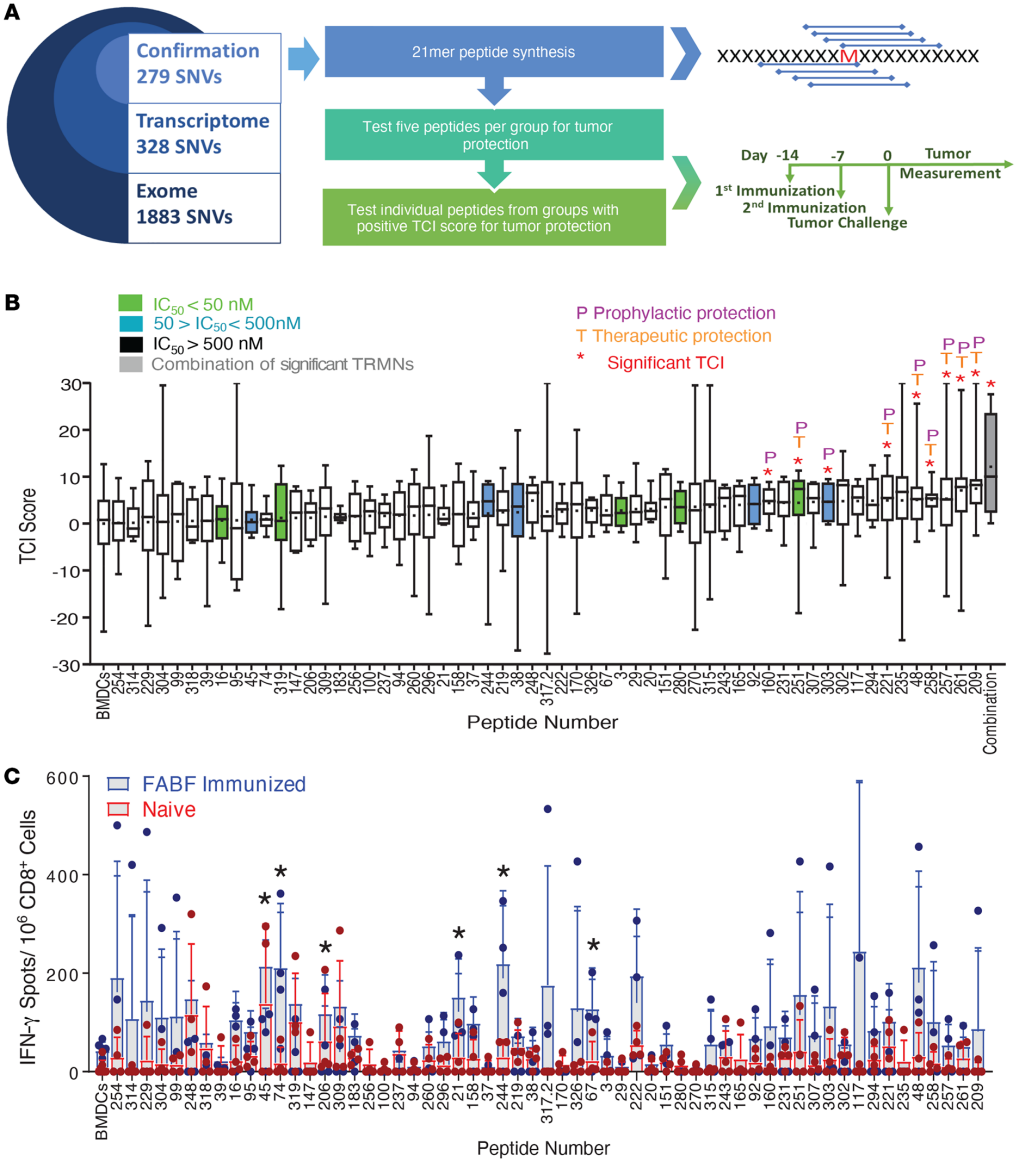
Unbiased identification of TRMNs. (**A**) All experimentally confirmed SNVs of the MC38-FABF tumor, and screening strategy for tumor rejection. (**B**) Box-and-whisker plot representing the tumor control index (TCI) scores (9) for 58 of all 279 peptides, represented by numbers on the *x* axis. The remaining 221 peptides elicited no tumor control and are not shown. The negative control (extreme left) consists of mice immunized with unpulsed BMDCs. Peptides that elicited significant tumor control are marked by asterisks. P and T indicate activity in prophylaxis and therapy. Combination of 9 positive peptides (TRMNs) is on the extreme right. The IC_50_ values for peptide–MHC I (K^b^/D^b^) were predicted using NetMHC 4.0; the values represent the highest predicted binder for each SNV or an experimentally verified precise neoepitope. Peptides are color coded by IC_50_ values as indicated in the box. *n =* 5–15 mice/group, except for the 9 active peptides (TRMNs), for which *n =* 20–50 mice per peptide. All peptides were tested at least 3 times; the 9 active peptides (TRMNs) were tested between 4 and 8 times each. (**C**) CD8^+^ (IFN-γ ELISpot) responses to peptides from **B** in MC38-FABF–immunized (blue bars) or naive mice (red bars) (*n =* 4 mice/group). To generate the box-and-whisker plots, data from every single mouse were entered. The box extends from the 25th to 75th percentiles, the middle line represents the median in each group, and the “+” represents the mean. The whiskers extend from the minimum to maximum value. Statistical analysis was conducted for peptides’ response against wells with no target. All peptides were tested at least 2 times. (**B** and **C**) Mean ± SD shown. *P < 0.05 by Student’s *t* test (**B**) or 2-way ANOVA (**C**).

The exome sequences from a murine colon cancer line MC38-FABF (C57BL/6J) were compared with the reference C57BL/6J exome, and 1883 single-nucleotide variants (SNVs) were identified (Figure 1A and Supplemental Data 1; supplemental material available online with this article; https://doi.org/10.1172/JCI142823DS1). Of these, genes expressing 328 SNVs were detected in the tumor transcriptome. Of these, 279 SNVs (85%) were validated by Sanger sequencing. For an unbiased analysis of the antitumor activity and CD8^+^ immunogenicity of every validated SNV, 279 peptides were synthesized. The peptides were 21 amino acids long, with the mutation at the center of the peptide, so as to include all putative 8- to 11-mers that may be presented by MHC I.

The 279 peptides were randomly grouped into 56 pools of 4 to 5 peptides per group. Mice were immunized prophylactically with each group using bone marrow–derived DCs (BMDCs) as adjuvants (8). In order to prevent discrepancies in peptide uptake due to competition, BMDCs were separately pulsed with each individual neoepitope from the group. The individually pulsed BMDCs were then pooled and used to immunize mice. Unpulsed BMDCs were used as a control. All mice were challenged with 30,000 MC38-FABF cells and serial tumor measurements were converted into tumor control index (TCI) scores (ref. 9 and Supplemental Figure 1A). A higher TCI indicates better tumor rejection, tumor stabilization, or inhibition of tumor growth. Almost half the groups (24/56 groups or 42%) had a positive TCI score (Supplemental Figure 1A), even though only 2 groups showed statistically significant activity. Regardless of statistical significance, 120 peptides within the 24 active groups were individually tested for their capacity to elicit tumor control (Figure 1B). Of the 120 peptides, 48% (58 peptides) showed a positive TCI score. Tumor sizes within individual groups showed considerable variation, as seen in Figure 1B. Notwithstanding this variation, about 20 peptides showed statistically significant or nearly significant TCI scores. These candidate active peptides were then tested in 15 to 40 mice per peptide; 9 peptides showed reproducible and statistically significant tumor control and are referred to as TRMNs (Figure 1B). Combination of the 9 TRMNs statistically outperformed 3 of the 9 bona fide TRMNs and trended toward better tumor control compared with the other 6 (Figure 1B and Supplemental Figure 1C). In addition to prophylactic immunization, the 9 TRMNs were tested for their ability to elicit therapeutic benefit in mice bearing preexisting tumors. Seven out of 9 TRMNs were active in therapy (Figure 1B).

NetMHC 4.0 (http://www.cbs.dtu.dk/services/NetMHC/) was used to predict the binding affinity (IC_50_) of each SNV-encoded peptide for K^b^ and D^b^ alleles. The 58 peptides in Figure 1B are color coded for the range of their affinities for K^b^ or D^b^. Surprisingly, 8 of 9 TRMNs had an IC_50_ value greater than 500 nM (Supplemental Table 1). Only 1 peptide (peptide 251) showed a strong-binding IC_50_ value of 14.8 nM. Remarkably, if all 279 candidate peptides were screened for tumor control based on high binding to MHC I (low IC_50_ values), 8 of the 9 TRMNs would have been excluded.

The reactivity of CD8^+^ T cells from mice immunized with irradiated MC38-FABF tumor cells to each of the 279 peptides was tested, and 4 peptides showed a statistically significant CD8^+^ response (Supplemental Figure 1B). The CD8^+^ reactivity for the 58 peptides corresponding to those in Figure 1B is shown in Figure 1C; 6 of 58 peptides elicited a statistically significant positive CD8^+^ response. None of the TRMNs showed a statistically significant CD8^+^ response, and none of the 6 peptides that showed CD8^+^ reactivity elicited tumor control. CD8^+^ T cells from mice immunized with the 2 strongest TRMNs, FAM171b^MUT^ and COX6a2^MUT^, were also tested for cytotoxicity against MC38-FABF; however, no cytotoxicity was observed.

**Figure 2:**
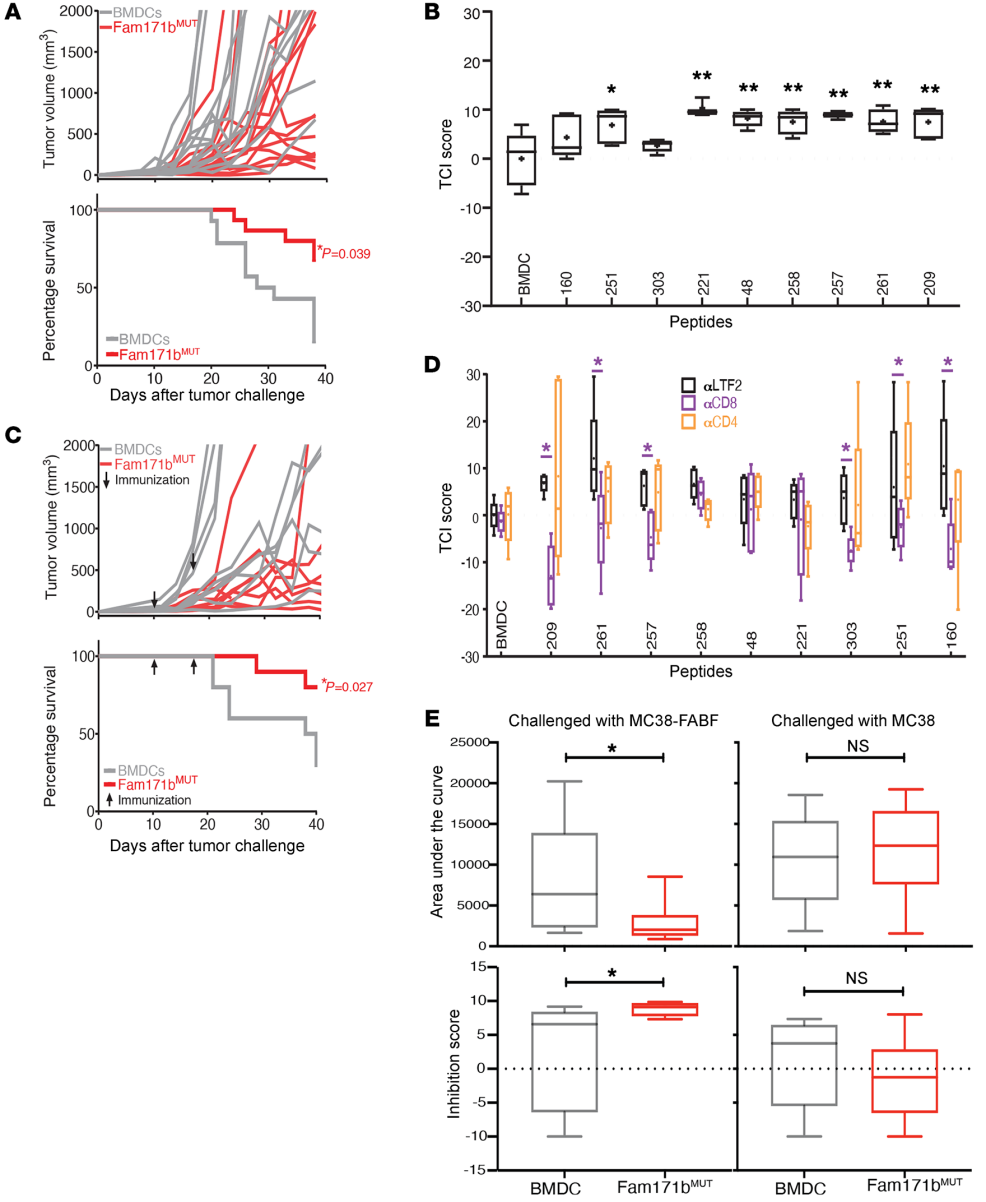
Characterization of the activity of TRMNs. (**A**) Tumor growth curves (top) and percentage survival (bottom) of mice immunized prophylactically with FAM171b^MUT^ (red) or unpulsed BMDCs (gray). Each line shows tumor volume for 1 mouse. The experiment was done 2 times (*n =* 10 and *n =* 5). (**B**) TCI scores of mice treated with each of the 9 TRMNs on days 0 and 7 after tumor challenge. *n =* 10 mice/group. The experiment was done twice. (**C**) Tumor growth curves (top) and percentage survival (bottom) of mice treated on days 10 and 17 after tumor challenge (indicated by arrows) with FAM171b^MUT^ (red) or unpulsed BMDC (gray), *n =* 10 mice/group. The experiment was done twice. (**D**) TCI scores of mice immunized with the 9 TRMNs and depleted of CD8^+^ (purple) or CD4^+^ cells (orange) or treated with an isotype control antibody (αLTF2) (black). The experiment was done twice. *n =* 5 mice/group. (**E**) Mice (*n =* 15) were immunized with unpulsed or FAM171b^MUT^-pulsed BMDCs. Five days later, CD8^+^ cells were isolated from the inguinal and popliteal lymph nodes. Two million CD8^+^ T cells were adoptively transferred into 9 mice/group. Mice were challenged with MC38-FABF on the right flank and MC38 on the left flank. Tumor growth was monitored. Data represent area under the curve (top) and growth inhibition (bottom) in mice that received T cell transfers from unpulsed BMDC-immunized mice (gray) or FAM171b^MUT^-immunized mice (red). *P < 0.05; **P < 0.01 by log-rank (Mantel-Cox) test (survival plots in **A** and **C**), Student’s *t* test (**B** and **E**), or 2-way ANOVA with Tukey’s multiple-comparison test (**D**). Box-and-whisker plots were generated as in Figure 1.

The activity of one of the strongest TRMNs, FAM171b^MUT^ (peptide 209 in Figure 1, B and C), is shown in detail (Figure 2A). There was a wide range of variation in the tumor growth curves in control and TRMN-immunized mice, as expected. For this reason, the tumor rejection experiments were carried out in large numbers of mice (*n =* 10–40). Prophylactic immunization with FAM171b^MUT^ elicited significant tumor control as measured by tumor growth (*P* < 0.05) and by survival (*P* = 0.039) (Figure 2A). Similar data with the TRMN COX6a2^MUT^ are shown in Supplemental Figure 2B. Immunization with unmutated peptides did not elicit tumor growth control or better survival (Supplemental Figure 2, B and C). All 9 TRMNs were tested for efficacy when immunization was carried out on the day of tumor challenge and day 7 after tumor challenge rather than 14 days and 7 days before tumor challenge (as in Figure 2A and Supplemental Figure 2); 7 out of 9 TRMNs elicited significant benefit in this relatively advanced setting of disease (Figure 2B). The effect of therapeutic immunization with FAM171b^MUT^ on 10-day-old tumors, which were clearly visible and palpable, was tested. As seen in Figure 2C, tumors of all mice in both groups showed nearly identical growth at the beginning of therapy; however, mice treated with FAM171b^MUT^ showed a significant inhibition of growth (*P* < 0.05) and improved survival (*P* = 0.027, Figure 2C). CD8^+^ and CD4^+^ T cell dependence of the antitumor activity of each TRMN was tested by depletion of respective subsets in vivo; the activity of 6 of 9 TRMNs was clearly CD8^+^ T cell dependent; the data for the remaining 3 TRMNs were inconclusive (Figure 2D).

In light of the CD8 dependence of TRMN-elicited tumor immunity in the context of lack of activity of CD8^+^ T cells from TRMN-immunized mice in vitro in ELISpot and cytotoxicity assays, the activity of TRMN-elicited CD8^+^ T cells was tested in vivo in an adoptive transfer assay. Naive C57BL/6J mice were adoptively transferred with the CD8^+^ T lymphocytes isolated from mice that had been immunized with BMDCs alone or with BMDCs pulsed with the active TRMN FAM171b^MUT^. The recipient mice were challenged 1 day after the adoptive transfer on 1 flank with the tumor MC38-FABF that had the mutation, and on the other flank with the MC38 line that did not have the mutation. We observed (Figure 2E) that the growth of the MC38-FABF tumor was inhibited significantly, whereas the growth of the MC38 line that lacked the mutation was not inhibited. There was no activity in mice that received CD8 lymphocytes from mice immunized with unpulsed BMDCs.

**Figure 3:**
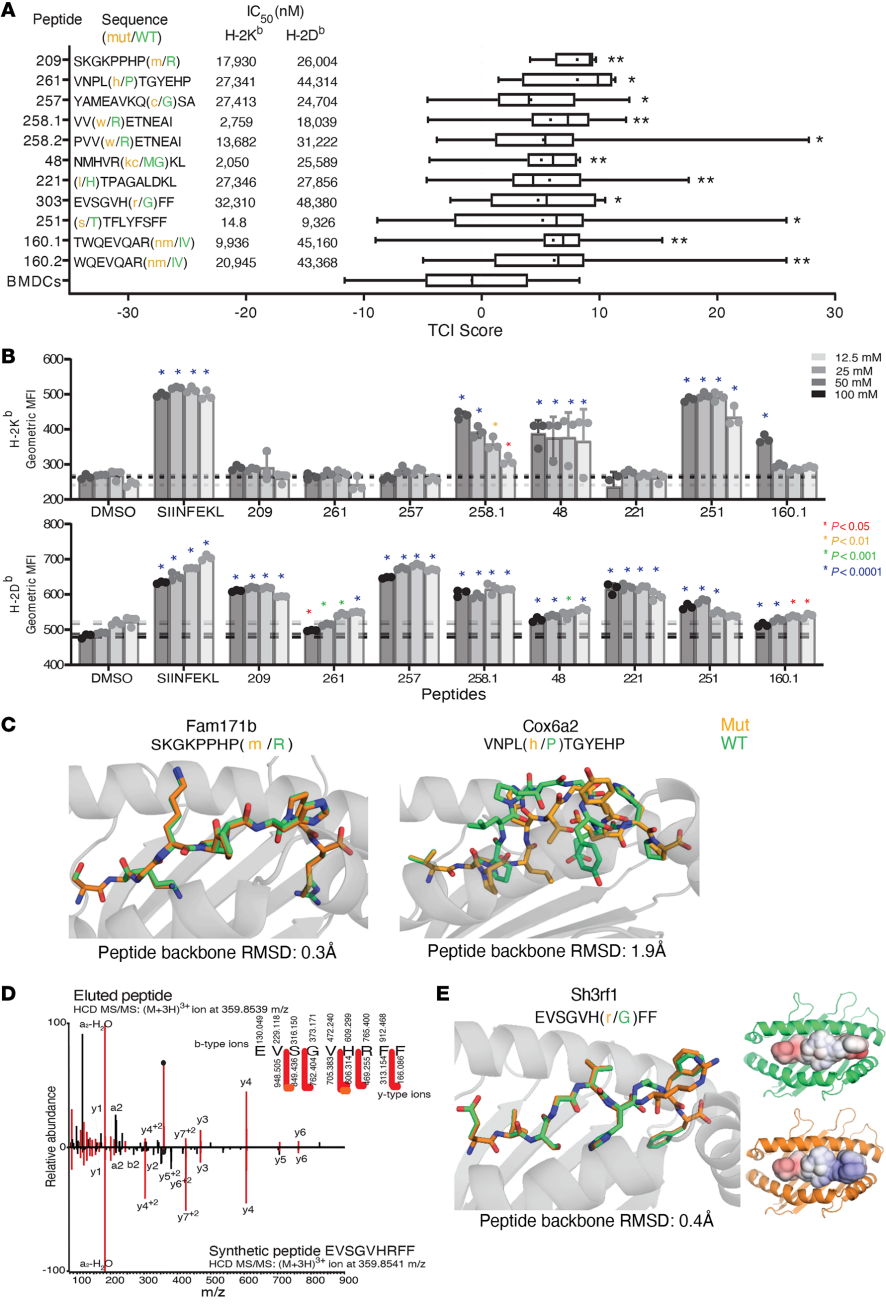
Definition of precise peptides for FAM171b and COX6a2 and their interaction with cognate MHC I alleles. (**A**) Sequences and binding affinities for K^b^ and D^b^ of the putative precise peptides of the 9 TRMNs (left); TCI scores of mice immunized with precise TRMN peptides, *n* = 15 mice/group (right). *P < 0.05, **P < 0.01 by Student’s *t* test. (**B**) Geometric MFIs of K^b^ (top) and D^b^ (bottom) of RMA-S cells pulsed with precise TRMN peptides. Data represent mean of triplicate values ± SD. *P < 0.05 by 2-way ANOVA. Each peptide was tested at least 2 times. (**C**) Structural models of binding of K^b^ with precise peptides of WT and mutant FAM171b, COX6a2. The WT is shown in green and the mutant in orange, with the MHC binding groove in gray. (**D**) MS/MS mirror plot displaying similarity of overall fragment ion coverage and relative abundances of identified fragment ions between a single-scan pulsed BMDC MS/MS (top pane) matched to sequence EVSGVHRFF and the single-scan MS/MS of the corresponding synthetic peptide (bottom pane). Fragment ions and neutral losses are labeled in both spectra, shared ions are shaded maroon, and singly charged (red arrows) and doubly charged ions (orange arrows) are annotated as observed for the pulsed BMDC peptide in the fragment ion coverage map. Ions represented by “•” denote those that fall within the prescribed isolation window. (**E**) Left: structural model of SH3RF1 bound to K^b^. The color scheme is as in **C**. APBS electrostatic surface potentials of mutant Sh3rf1 (top right) and WT Sh3rf1 (bottom right). Surface potentials are on a scale of –4.000 (blue) to +4.000 (red) k_B_Te_c_^–1^, or approximately 26.7 mV per 1.000 at 310 K. Box-and-whisker plots were generated as in Figure 1.

The experiments described thus far were carried out with 21-mer peptides. Next, the precise epitopes of the 9 TRMNs were identified. We tested the precise peptides that had the highest predicted binding affinities for K^b^ or D^b^ in tumor control assays, as in Figure 1B; the TCI of each peptide is shown (Figure 3A). Because Figure 3A shows data on tumor control, and not antigen presentation per se, the peptides most active in tumor control in Figure 3A were tested for their ability to pulse RMA-S cells in vitro and stabilize peptide–MHC I (pMHC I) complexes recognizable by allele-specific antibodies for K^b^ and D^b^. Each TRMN was observed to be presented by one or both alleles (Figure 3B). For the purpose of identification of the precise neoepitopes, the 2 assays (prediction by tumor rejection and stabilization of pMHC I complexes) yielded completely consistent results.

The TRMN SH3RF1^MUT^, on the other hand, presents a highly illustrative example of the lack of correlation between prediction and actual presentation and between MHC I–binding and tumor control activity. The long peptide that harbors the mutation in SH3RF1^MUT^ was interrogated for prediction of binding of a precise epitope of K^b^ or D^b^. Of the possible candidates, peptide VHRFFPTNF was predicted to bind K^b^ with the highest affinity of an IC_50_ of 332 nM. Interestingly, we were able to identify the precise neoepitope within the SH3RF1^MUT^ long peptide by pulsing the long peptide onto BMDCs and eluting presented epitopes from the MHC I of the BMDCs (Figure 3D). The presented neoepitope, as identified by mass spectrometry (MS), turned out to be EVSGVHRFF, which has a predicted binding affinity (for K^b^) of 32,310 nM, 2 orders of magnitude lower than the predicted affinity of the strongest binding peptide VHRFFPTNF. This observation underscores the lack of correlation between affinity for MHC I and tumor control, as seen in Figure 1B.

### Molecular modeling of MHC class I–peptide interaction.

We modeled the structures of selected TRMNs and their WT counterparts. The data on 3 TRMNs, COX6a2^MUT^, FAM171b^MUT^, and SH3RF1^MUT^ bound to K^b^, are shown since each suggests a different manner of immunogenicity of the TRMN. Models were generated using stochastic, flexible-backbone protein-modeling protocols (10–12). The proline to histidine mutation at position 5 in the COX6a2^MUT^ was predicted to yield a neoepitope with a substantially different conformation than the WT peptide (Figure 3C). The neoepitope was predicted to adopt a more compact backbone conformation than the WT peptide in the K^b^ binding groove, decreasing the total solvent-accessible surface area (SASA) by 19% (from 484 Å^2^ to 390 Å^2^) and hydrophobic SASA by 28% (from 323 Å^2^ to 232 Å^2^). This substantial difference in peptide conformation can explain the immunogenicity of the COX6a2^MUT^, in that a T cell population tolerant of the WT peptide would encounter a peptide-MHC complex with substantially different surface properties. Unlike COX6a2^MUT^, the arginine-to-methionine mutation at position 9 in the FAM171b^MUT^ was predicted to have no significant impact on peptide conformation when bound to K^b^ (Figure 3C). The immunogenicity of FAM171b^MUT^ may stem instead from the more stable presentation of the neoepitope than its WT counterpart, as in the conclusion drawn by Duan et al. (6) that a stabilizing mutation at a primary anchor position can lead to an immunogenic neoepitope by reducing the entropic cost associated with T cell receptor (TCR) binding and thus enhance receptor affinity. In the SH3RF1^MUT^ peptide, the modeling predicted that the glycine-to-arginine mutation at position 7 of the peptide would alter peptide conformation only slightly (Figure 3E). However, compared with the WT peptide, the exposed surface presented to TCRs at the C-terminal end of the neoepitope was considerably altered because of the additional bulk of the arginine side chain. The change increased exposed SASA by 17% (from 322 Å^2^ to 378 Å^2^) and more importantly, resulted in the exposure of a positive charge, again contributing to a peptide whose presented surface would appear substantially different to a TCR (Figure 3E).

To examine the conformational stability of these static models, we performed molecular dynamics simulations on each of the final models described above. Each model was simulated for 300 ns in explicit solvent. In general, all peptides retained their conformations throughout simulation. Only slight perturbations occurred in backbone dihedrals and side chain rotamers, supporting the conclusions drawn from the static structural models (Supplemental Figure 3, A and B). The FAM171b^MUT^ peptide was indeed more stable than its WT counterpart in the K^b^ binding groove, as hypothesized, at both C- and N-termini. A new insight gleaned from the molecular dynamics simulations was substantially lower conformational sampling by SH3RF1^MUT^ than WT, likely owing to the glycine-to-arginine mutation. Building on the conclusions drawn from the static models, this difference in flexibility would serve to amplify the differences between the WT and mutant peptides (Supplemental Figure 3C).

### CD8^+^ TILs of FAM171b^MUT^-immunized mice have a stem-like early dysfunctional phenotype.

**Figure 4:**
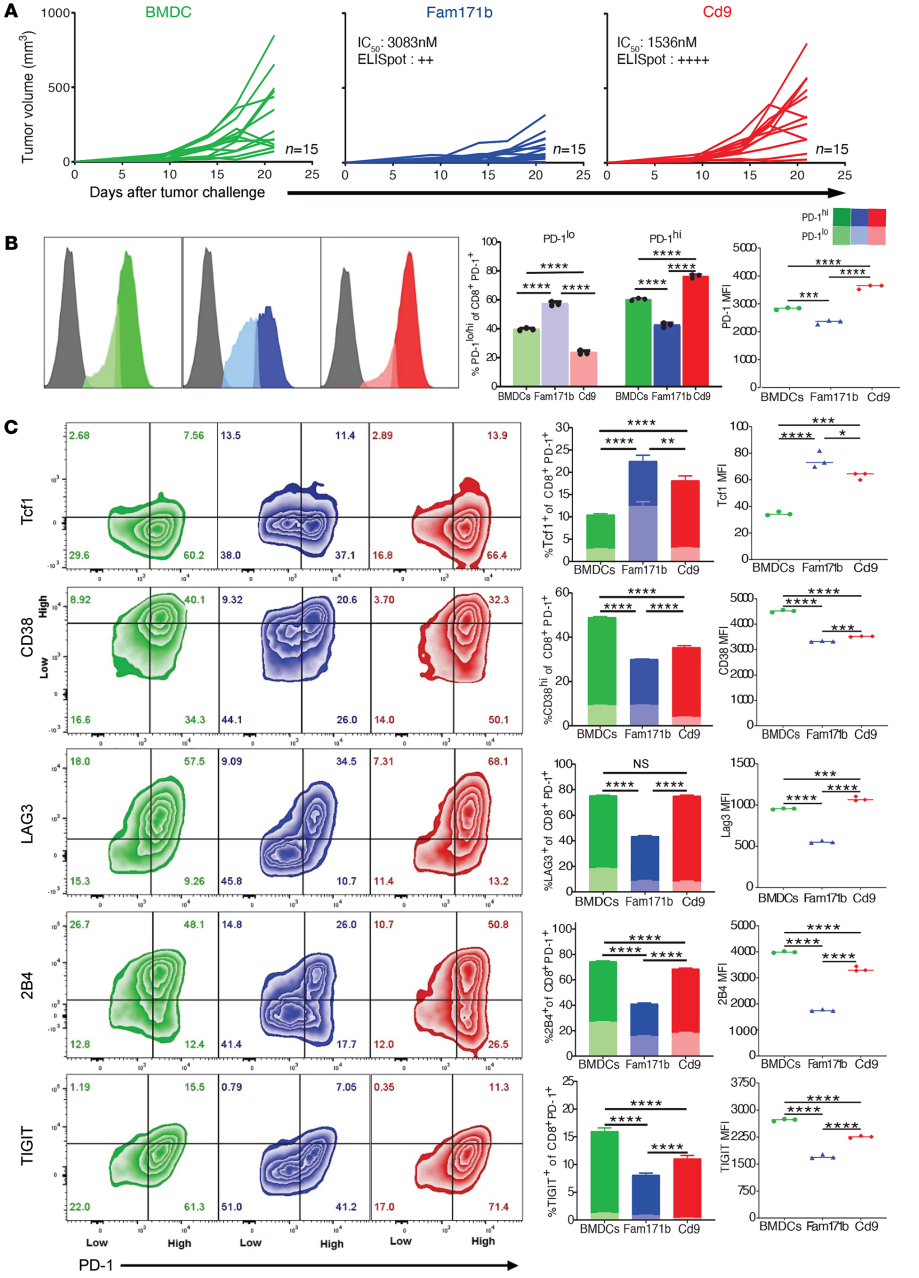
Phenotypes of CD8^+^ TILs from mice immunized with a TRMN and a non-TRMN. Mice (*n =* 15 mice per group) were immunized with unpulsed BMDCs (green) or BMDCs pulsed with peptides FAM171b^MUT^ (a TRMN, blue) or Cd9^MUT^ (a non-TRMN, red) and challenged with MC38-FABF. Tumors were harvested on day 25 after tumor challenge and CD8^+^ TILs isolated. (**A**) Tumor growth of mice immunized with each group. IC_50_ values for cognate alleles and IFN-γ ELISpot response of CD8^+^ T cells from spleens of MC38-FABF–immunized mice are indicated for each peptide (0–50 spots/10^6^ CD8^+^ cells = ++, >140 spots/10^6^ CD8^+^ cells = ++++). (**B**) MFI of PD-1 in CD8^+^ TILs (left); bar graph representing percentage of PD-1^lo^ and PD-1^hi^ cells (middle; data represented as mean ± SD with individual points); quantification of MFI of PD-1 (right). *n =* 5 pooled mice per group, 3 technical replicates. (**C**) Flow cytometry contour plots with indicated markers in CD8^+^PD-1^+^ (low and high) TILs (left) with respective stacked bar graphs representing percentage of cells (middle) and quantification of MFI (right). Data represented as mean ± SD; *n =* 5 pooled mice per group, 3 technical replicates. *P < 0.05, **P < 0.01, ***P < 0.001, ****P < 0.0001 by ANOVA with Tukey’s multiple-comparison test (**B** and **C**). The data are representative of 3 independent experiments.

Recent studies (13–16) have shown that the effective CD8^+^ T cell response in the context of chronic antigen exposure is mediated by CD8^+^ T cells that have a stem-like early dysfunctional phenotype, as opposed to a terminally exhausted phenotype. The tumor-infiltrating lymphocytes (TILs) from the mice immunized with a TRMN (FAM171b^MUT^) and a non-TRMN (Cd9^MUT^) were characterized in this regard. Mice were immunized with FAM171b^MUT^-pulsed BMDCs and challenged with MC38-FABF cells; these mice showed significant tumor control (Figure 2A and Figure 4A) and a weak and statistically insignificant IFN-γ ELISpot response (Figure 1C and Figure 4A). As controls, mice were immunized with unpulsed or Cd9^MUT^-pulsed BMDCs and challenged with MC38-FABF cells. Cd9^MUT^ (peptide 244 in Figure 1C and Figure 4A) is a mutant peptide that did not elicit tumor control but elicited statistically significant IFN-γ ELISpot CD8^+^ T cell response. Flow cytometric analysis of CD8^+^ TILs isolated from 25-day-old tumors from the 3 groups revealed that TILs from FAM171b^MUT^-immunized mice had a unique PD-1^lo^ population that was nearly absent in both control groups (Figure 4B). This difference in PD-1 expression could be seen in the proportion of PD-1^lo^ TILs as well as the MFI of total PD-1 expression among the 3 groups of mice (Figure 4B). TILs from all groups contained a PD-1^hi^ population, although the proportion of this population was lowest in TILs of FAM171b^MUT^-immunized mice (Figure 4B). The TILs were also analyzed for expression of Tcf1, CD38, LAG3, 2B4, and TIGIT because their expression profiles have been used to describe functional states of TIL, including less dysfunctional/plastic and severe dysfunction (17). TILs from FAM171b^MUT^-immunized mice showed a higher proportion of Tcf1^+^ cells specifically within the PD-1^+^ population (Figure 4C). It is also evident from the flow contour plot in Figure 4C that the TILs from the FAM171b^MUT^-immunized group contained a Tcf1^+^PD-1^lo^ population that was nearly absent in the TILs of the 2 control groups. The MFI of Tcf1 expression by the TILs of the 3 groups was consistent with this interpretation. The TILs of FAM171b^MUT^-immunized mice showed a lower proportion of CD38^hi^ cells as well as lower overall expression of CD38 as measured by MFI (17). The TILs of FAM171b^MUT^-immunized mice also showed a lower proportion of cells expressing the coinhibitory receptors LAG3, 2B4, and TIGIT (as well as significantly reduced MFI of these markers) than the TILs of control mice.

**Figure 5:**
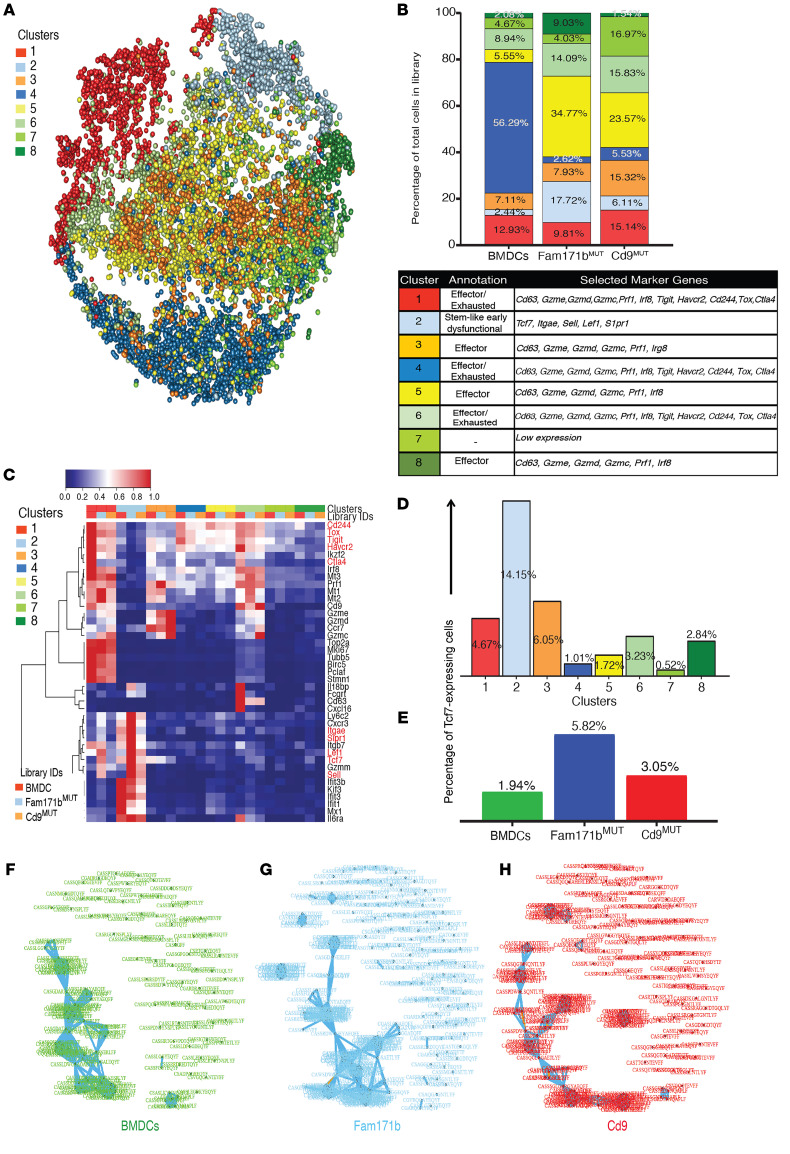
Single-cell RNA-Seq analysis of CD8^+^ PD-1^+^ TILs from mice immunized with a TRMN and a non-TRMN. Mice (*n* = 3 per group) were immunized with unpulsed BMDCs or BMDCs pulsed with peptides FAM171b^MUT^ (a TRMN) or Cd9^MUT^ (a non-TRMN) and challenged with MC38-FABF. Tumors were harvested on day 25 after tumor challenge and live CD8^+^PD-1^+^ TILs isolated by FACS and sequenced by scRNA-Seq. Approximately 4400 CD8^+^PD-1^+^ TILs were analyzed in each library. (**A**) Three-dimensional t-SNE plot showing clustering based on top average TF-IDF genes. (**B**) Top: composition (distribution) plot showing percentage of cells in the 8 clusters along with respective annotations in unpulsed BMDCs, FAM171b^MUT^, and Cd9^MUT^ libraries; bottom: table showing cluster annotation based on selected markers. (**C**) Summary heatmap of selected differentially expressed genes (threshold of differential expression as defined in Methods). (**D** and **E**) Percentage of Tcf7-expressing cells in each of the 8 clusters (**D**) or in each of the 3 libraries as indicated (**E**). (**F**–**H**) Cluster results of applying GLIPH to the TCRs of each library as indicated. Each node is a TCR and each edge between the TCRs indicates the GLIPH-predicted shared specificity. Blue edges indicate shared local motif and orange edges indicate shared global similarity.

CD8^+^PD-1^+^ populations of TILs from the 3 groups were also analyzed by single-cell RNA sequencing (scRNA-Seq). The combined TILs from the 3 groups of mice resolved into 8 clusters based on their transcriptional profiles (Figure 5A). Figure 5B shows the relative proportion of the 8 cell types (clusters) among the TILs from the 3 groups of mice. Each of the 8 clusters was annotated based on the expression of select markers and differentially expressed genes (Methods and Supplemental Data 3 and 4). Based on this annotation, clusters 1, 4, and 6 expressed markers of effector as well as exhausted phenotypes (Figure 5, B and C), whereas cluster 3 expressed markers associated only with an effector phenotype. Cluster 7 had overall low expression of genes and hence could not be identified. Cells in clusters 2, 5, and 8 showed expanded proportions in TILs of FAM171b^MUT^-immunized mice as compared with the non-TRMN or BMDC-alone groups. Clusters 5 and 8 constituted effector cells (based on expression of Cd63, Gzme, Gzmd, Gzmc, Prf1, and Irf8; Figure 5, B and C). Cluster 2 comprised cells with a stem-like early dysfunctional phenotype (13–16), as seen by an upregulated expression of Tcf7 (Tcf1), Itgae (CD103), Sell (CD62L), Gzmm, Lef1, and S1pr1 (Figure 5, B and C, and Supplemental Figure 5). Cells in cluster 2 were also observed to express lower levels of markers associated with exhaustion such as Tigit, Havcr2, Cd244, Tox, and Ctla4. Within cluster 2, the differentially upregulated genes were expressed at a higher level in TILs from FAM171b^MUT^-immunized mice than the other 2 control groups (Figure 5C and Supplemental Figure 5). Tcf7 expression among the 3 groups was analyzed further (Figure 5, D and E). Consistent with the higher expression of Tcf7 in cluster 2 (Figure 5C and Supplemental Figure 5), this cluster was observed to have the highest proportion of Tcf7-expressing cells among the combined TIL population of the 3 groups (Figure 5D). By overall expression of Tcf7 among the TILs of the 3 groups of mice, Tcf7 was upregulated in FAM171b^MUT^-immunized mice as compared with the other 2 control groups (Figure 5E).

TCRs in the TILs of the 3 groups of mice were characterized using grouping of lymphocyte interactions by paratope hotspots (GLIPH) analysis. This analysis grouped together the TCRs into specificity groups based on the similarity of the CDR3 regions of the TCRs (see Methods) and showed that TILs of FAM171b^MUT^-immunized mice contained a higher number of specificity groups (9 groups) than those of BMDC-immunized mice (3 groups) or Cd9^MUT^-immunized mice (6 groups). In TILs of FAM171b^MUT^-immunized mice, 129 distinct CDR3 sequences contributed to the largest specificity group, as opposed to 74 and 87 distinct CDR3 sequences in TILs of naive or Cd9MUT-immunized mice. This observation is consistent with increased clonality of antitumor TCRs in TILs of Fam171bMUT–immunized mice (Figure 5, F–H). The largest specificity group in BMDC-immunized mice also included the most frequent clone (highest proportion of T cells with the same CDR3 sequence). In contrast, the most frequent clone in FAM171b^MUT^- and Cd9^MUT^-immunized mice did not belong to the largest specificity groups, suggesting that a high frequency of a given clone does not necessarily correlate with the size of a specificity group.

### A model for enriching for TRMNs from data in silico.

**Figure 6:**
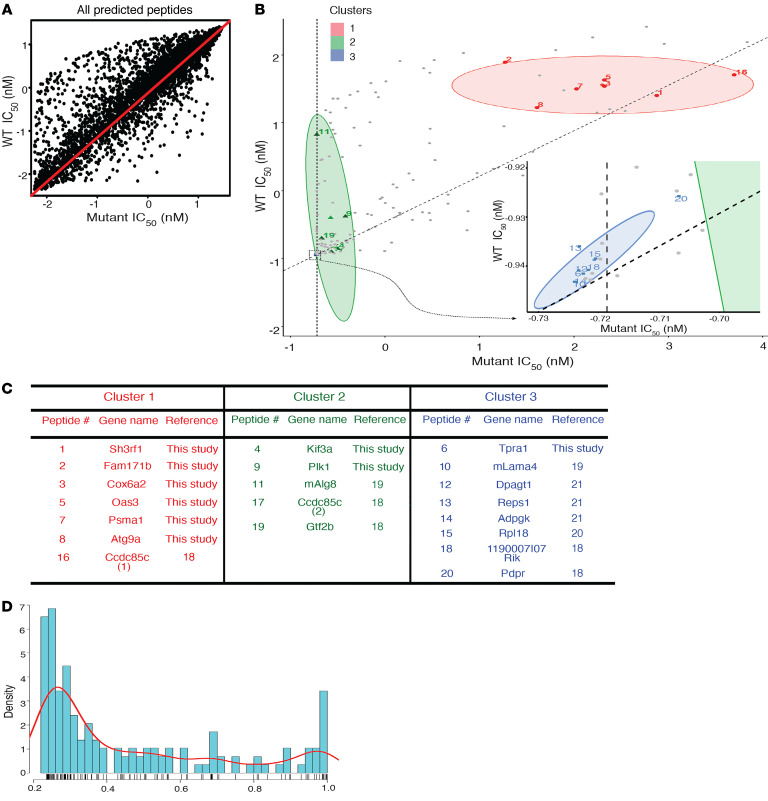
Defining TRMNs with novel characteristics. (**A**) Scatter plot of the normalized (scaled and centered) values (for every potential precise peptide for each SNV tested) of mutant IC_50_ (nM) on the *x* axis versus the WT IC_50_ (nM) on the *y* axis. The red diagonal represents equal IC_50_ values for mutant and WT or DAI value of 0 in scale. (**B**) Plot shows the bivariate scatter plot of the normalized reference and mutant IC_50_ values of all the peptides; the TRMNs group in 3 clusters: red circles in cluster 1 (7 peptides), green triangles in cluster 2 (5 peptides), and blue squares in cluster 3 (9 peptides). All non-TRMNs are in gray. Inset: zoomed-in illustration of cluster 3. (**C**) Table listing all TRMNs in the 3 clusters. (**D**) Plot showing the density of scaled mutant IC_50_ values of all TRMN and non-TRMN neoepitopes of MC38-FABF.

In addition to considering the affinity of a neoepitope for an MHC allele, we considered the affinity of the corresponding unmutated peptide for the MHC allele (6). When neoepitopes, which are in effect altered peptide ligands, are presented by the MHC, the affinity for these neoepitopes for an MHC allele can be the same, higher, or lower than the corresponding affinity for the unmutated epitope. In Figure 6A, where the affinities for all possible mutated epitopes and their unmutated counterparts are plotted along 2 perpendicular axes, the diagonal represents an equal affinity of the 2 counterparts for MHC. Figure 6A shows a scatter plot of the normalized (scaled and centered) values of mutant IC_50_ (nM) on the *x* axis and the reference IC_50_ (nM) on the *y* axis. Generally speaking, most points have similar affinities for unmutated and mutated counterparts, resulting in the points being distributed symmetrically around the diagonal line. The differential agretopic index (DAI), a measure for difference-from-self, for neoepitopes on the diagonal (red line) is zero. Any candidate neoepitopes that fall below the diagonal have a negative DAI, and those above the diagonal have a positive DAI. In Figure 6B, we plotted the data (normalized as described in Methods) for the 9 TRMNs defined in Figure1B, as well as those previously published by us (18) and others (19–21). The plot shows the bivariate scatter plot of the normalized reference and mutant IC_50_ values of the peptides, with points in gray representing all negatives, and positives in various colors are grouped in 3 clusters: red circles in cluster 1 (7 peptides), green triangles in cluster 2 (5 peptides), and blue squares in cluster 3 (9 peptides). The number of clusters was determined by analyzing the Bayesian information criterion (BIC) and the clusters/ellipses were fit using model-based clustering based on parameterized finite Gaussian mixture models using the reference and alternative IC_50_ values as features. The dashed vertical line in Figure 6B indicates IC_50_ = 50 nM. The TRMNs in the individual clusters are listed in Figure 6C. A number of patterns are clearly discernible in the 3 clusters: (a) cluster 3 contains TRMNs with high affinity for MHC I (IC_50_ values between 2 and 157 nM) and includes TRMNs published by us in this study and (18) as well as others (19–21); (b) cluster 2 contains TRMNs with a broader range of affinities for MHC I, with IC_50_ values between 44 and 2,759 from this study and others (18, 19); (c) cluster 1 is entirely different from all previous TRMNs and consists of 7 TRMNs with extremely low MHC I–binding affinities identified in this study (IC_50_ values of 17,930, 20,945, 24,704, 27,341, 27,346, and 32,310 nM) as well as a TRMN previously published by us (18) (39,661 nM). Thus, cluster 1 represents a potentially novel space for the existence of TRMNs, which has been revealed solely on the basis of our unbiased analysis of TRMNs.

The proportion of TRMNs and non-TRMNs within each cluster was calculated and compared. Since the true number of non-TRMNs were identified only in this study, only the data from MC38-FABF as shown here were used in this calculation. Cluster 1 contained 6 TRMNs and 5 non-TRMNs (55% TRMNs), cluster 2 contained 2 TRMNs and 46 non-TRMNs (4% TRMNs), and cluster 3 contained 1 TRMN and 6 non-TRMNs (14% TRMNs). This calculation was also performed in a manner that all neoepitopes (both inside and outside the boundaries of the plotted ellipses) were forced to choose a cluster using the fitted mixture model estimation of the clusters described in Figure 6C. By this unbiased analysis, cluster 1 contained 6 TRMNs and 35 non-TRMNs (15% TRMNs), cluster 2 contained 2 TRMNs and 89 non-TRMNs (2% TRMNs), and cluster 3 contained 1 TRMN and 12 non-TRMNs (8% TRMNs). The enrichment of cluster 1 in TRMNs is also emphasized by the fact that among all neoepitopes, cluster 1 is located in the lower density range of the mutant IC_50_ values described in Figure 6D, which is underrepresented compared with the highest global density (high-affinity range). These considerations demonstrate that the newly defined cluster 1 was the most highly enriched in TRMNs.

In order to test the generality of cluster 1 neoepitopes as TRMNs, we needed tumor rejection data where a sufficient number of TRMNs from a single tumor distinct from MC38-FABF had been identified. The published data (6) on neoepitope-mediated tumor rejection of a CMS5 tumor of a different haplotype and different tissue type (BALB/c fibrosarcoma vs. the C57BL/6J-derived colon carcinoma MC38-FABF) were the only ones to fit this criterion. In this tumor, the neoepitopes predicted by the high-affinity–binding method, which is the core of all current methods, had completely failed to elicit tumor immunity; however, 6 neoepitopes with poor affinity for MHC I had been shown to elicit tumor rejection (6). We overlapped the NetMHC4 predicted mutant and WT IC_50_ values of the 6 CMS5 TRMNs with the scatter plot from Figure 6B, preserving the same model parameters and scaling used to define the clusters described in Figure 6B. With the exception of one outlier, all of the superimposed scaled IC_50_ values of the CMS5 TRMNs fell within the boundaries or closely adjacent to cluster 1 (Supplemental Figure 6), which was learned solely using MC38-FABF data. Clearly, the cluster 1 space needs further refinements, including larger numbers of TRMNs, as well as consideration of additional characteristics of TRMNs and non-TRMNs.

## Discussion

Our study reports an unbiased analysis of the capacity of all confirmed SNVs in a tumor to elicit tumor rejection and CD8^+^ T cell response. Our results generated in one tumor, and validated in another antigenically distinct tumor, challenge 2 key dogmas in the field and clarify several aspects of the identity and activity of the TRMNs. One dogma is that a high-binding-affinity MHC I–peptide interaction is a requirement for antitumor activity. This dogma is based on established observations that such high affinity (better binding than an IC_50_ value of 50–100 nM) is critical for definition of viral epitopes that elicit CD8^+^ T cells (1). Our results showed definitively that this is not the case for TRMNs. Nine neoepitopes out of nearly 300 tested, or about 3% of the total, elicited measurable tumor rejection, and 8 of 9 had predicted affinities between IC_50_ values of 2000 nM and 33,000 nM. Two previous studies (19, 21) have reported antitumor activity of high-affinity neoepitopes, and one previous study has reported the same for low-affinity neoepitopes (6). Nelson and colleagues have reported a complete absence of antitumor activity among all the high-affinity MHC I–binding neoepitopes of a murine ovarian cancer (7). Our study reconciles these discordant observations and vastly expands the universe of TRMNs; using the criterion of high affinity for MHC I, 8 of 9 TRMNs identified in our study would have been discarded as candidates. Our results are supported by the retrospective analyses of the relationship between MHC I–neoepitope affinities and clinical outcomes in human studies (4, 5). Ghorani et al. (4) and Retch et al. (5) independently examined mutational and clinical outcome data from several thousand patients and, remarkably, concluded that presence or absence of high-affinity MHC-binding neoepitopes in tumors had no correlation with overall survival, progression-free survival, or T cell infiltration in tumors, while the presence of low affinity was powerfully correlated with all clinical endpoints in melanoma and lung cancers (4) and in 27 different tumor types (5).

The second dogma has to do with measurement of CD8^+^ T cell responses. It is an established fact that CD8^+^ T cells (among other immune elements) are essential for a successful antiviral and antitumor activity. This fact has generated a dogma: that the CD8^+^ T cells as measured contemporarily are true surrogate markers of antitumor CD8^+^ activity in vivo. This dogma persists in spite of the observations in mice (22–24) and in humans (25–27) that there is little correlation between measurable CD8^+^ T cell responses and clinical activity. The tumor immunity elicited by most of the 9 TRMNs identified here is CD8^+^ T cell dependent, as observed by the abrogation of immunity by depletion of CD8^+^ cells as well as by successful adoptive transfer of CD8^+^ T cells from TRMN-immunized mice to naive mice. Yet, ELISpot, cytotoxicity, or FACS assays, the current standards of measurement of CD8^+^ T cell activity, failed to detect significant CD8^+^ T cell response to any of these TRMNs. Lower precursor frequencies or tighter regulation of anti-TRMN responses (which are anti–altered-self responses) may contribute to this apparent discrepancy. It is also conceivable that the anti–TRMN CD8^+^ T cells manifest their antitumor activity by mechanisms other than direct action on tumor cells. Development of more sensitive assays such as those based on quantitation of TCR clones may also address this disparity between CD8^+^ T cell–dependent responses in vivo and lack of a CD8^+^ T cell response in vitro.

A characteristic of the anti–TRMN CD8^+^ T cells that can be measured ex vivo is the stem-like early dysfunctional phenotype (17). Plasticity of T cells (defined by higher levels of Tcf1 and lower levels of PD1, CD38, CD101, CD39, and TIGIT) has emerged as a significant factor in their function in vivo in viral and tumor models (14, 15, 17, 28). Our demonstration of such a phenotype in anti–TRMN CD8^+^ TILs is, we believe, the first such demonstration in endogenous CD8^+^ cells in a nontransgenic tumor. The stem-like early dysfunctional phenotype of the anti–TRMN CD8^+^ cells may also have a link with the low affinity of TRMNs for MHC I. The low affinity of TRMNs for MHC I may influence the phenotypes of T cells engaged by pMHC I complexes: (a) during cross-presentation, fewer antigen-presenting cells (APCs) present the low-affinity peptide (as compared with a high-affinity peptide), causing T cells to receive a signal through the TCR less frequently, and (b) on an APC that presents the low-affinity peptide, there will be fewer pMHCs on the cell surface that contain this peptide, resulting in a relatively lower avidity for T cells recognizing this pMHC on this cell. Both consequences will lead to a less-exhausted T cell phenotype (29–32).

There are a number of estimates about the frequency of TRMNs among all potential neoepitopes (6, 19, 21, 33, 34). These estimates, which range from 0.1% to 1%, are based on high affinity of neoepitopes for MHC and/or the proportion of neoepitopes against whom a CD8^+^ T cell response is detected. Since the present study is, to our knowledge, the only analysis of the definition of true TRMNs among all candidate neoepitopes, our conclusion that TRMNs constitute more than 3% of candidate neoepitopes is significant. Since our analysis covers only SNVs and does not take into consideration INDELS and other somatic variations, this number represents an underestimate of the true proportion of TRMNs. These considerations have profound consequences for our aspirations in human cancer immunotherapy.

## Methods

### Mice and tumor cell lines.

The C57BL/6J (6- to 8-week-old females) were purchased from The Jackson Laboratory. A chemically induced murine tumor cell line in the C57BL/6J background known as MC38-FABF was used as the primary tumor model for this extensive study. The MC38-FABF tumor cell line was provided by Alan Frey at New York University Langone Medical Center.

### Sample preparation for exome and RNA-Seq and bioinformatic analysis.

The exome and transcriptome of the MC38-FABF cell line were sequenced as described previously (6). Sequencing was performed following the newer version of the Epi-Seq pipeline as previously published by us (6). Exome and RNA-Seq reads were aligned to the mm10 mouse reference genome using HISAT2 (35). SNVs were called using the somatic variant caller version of SNVQ (36). The list of SNVs was generated for those mutations with both exome and RNA coverage for each SNV position. The EpitopeFinder tool of Epi-Seq then produced reference and alternative peptide sequences with predicted MHC I binding affinities and DAI (6) scores for called SNVs. Gene expression estimation from RNA-Seq data was performed by using IsoEM2 algorithm (37).

### Peptide synthesis.

Peptides were custom made with a purity of more than 90% (JPT and Genscript) and dissolved in DMSO at a final concentration of 20 mM.

### Generation of BMDCs and neoepitope vaccine preparation.

BMDCs were generated as per Inaba et al. (38) and were pulsed with 100 μM peptide for approximately 2 hours at 37°C, and then washed and resuspended in RPMI 1640.

### Immunization with neoepitopes and tumor challenge.

For prophylactic immunization, mice were immunized as described. Anti–CTLA-4 antibody was continued every 3 days until termination of the experiment. Mice were challenged with 30,000 tumor cells intradermally. Tumor volumes were measured using the Biopticon TumorImager. Tumor control indices were calculated for every experiment as described by Corwin et al. (9).

For therapeutic immunization, mice were challenged as above, and then immunized as before on day 0 or day 10 after tumor challenge along with anti–CTLA-4 (75 μg/ mouse, clone 9D9, Bio X Cell). A second immunization and anti–CTLA-4 antibody was administered 7 days later. Anti–CTLA-4 antibody was continued every 3 days until termination of the experiment.

### Intracellular IFN-γ assay by ELISpot.

As targets to stimulate the CD8^+^ cells, naive splenocytes pulsed with peptide were added to the wells. Plates were analyzed by ZellNet Consulting. We rated the magnitude of responses by mean spot numbers per million CD8^+^ cells: 5–10 (+), 11–20 (++), 21–50 (+++), 51–100 (++++), and more than 100 (+++++).

### Depletion of T cell subsets.

CD8^+^ cells were depleted using anti-CD8α rat IgG2b monoclonal antibody 2.43 (Bio X Cell). CD4^+^ cells were depleted using anti-CD4 rat IgG2b monoclonal antibody GK1.5 (Bio X Cell). Depleting antibodies were given in PBS (i.p.) 1 day before each immunization at 250 μg per mouse. Depletion was continued every 7 days for the duration of the experiment at 150 μg per mouse. The antagonistic antibody, anti–CTLA-4 (clone 9D9; Bio X Cell) was given at 75 μg, 7 days before and every 3 days after tumor challenge. The appropriate T cell subsets were depleted by more than 95%.

### Flow cytometry.

The antibodies for CD8α Pacific Blue (clone 53-6.7), CD8α APC-Cy 7 (clone 53-6.7), CD44 brilliant violet (clone IM7), PD-1 PCP-Cy5.5 (clone RMP1-30), PD-1 APC (clone RMP1-30), Tim3 APC (clone RMT3-23), Tim3 PCP-Cy5.5 (clone RMT3-23), Lag3 PE/Cy 7 (clone eBioC9B7W), CD62L APC-Cy7 (clone MEL-14), CD38 APC (clone 90), and CD38 PE/Cy7 (clone 90) were purchased from Biolegend. The antibodies for 2B4 PE-Cy7 (clone eBio244F4), 2B4 FITC (clone eBio244F4,) and TIGIT PCP–eFluor 710 were purchased from Thermo Fisher Scientific. The antibody for TCF1 Alexa Fluor 488 (clone C63D9), TCF1 PE (clone C63D9) was purchased from Cell Signaling Technology. For RMA-S experiments, antibodies for H2-K^b^ APC (clone AF6-88.5.5.3) and H2-D^b^ PE (clone 28-14-8) were purchased from Thermo Fisher Scientific. Flow cytometry was performed using a Miltenyi Biotec MACSQuant analyzer. Analysis was done using FlowJo software.

### MHC I stabilization on RMA-S cells.

Precise peptides of the TRMNs identified in Figure 3A were tested for their ability to bind H2-K^b^ or H2-D^b^ using RMA-S cells. RMA-S cells were cultured with the precise peptides at various concentrations at 37°C for 1 hour. The level of K^b^ or D^b^ complexes was tested by flow cytometry.

### Molecular modeling and dynamics of peptide-MHC complexes.

Structural modeling was performed as previously published (18). Briefly, Rosetta (10, 12, 39, 40) was used via PyRosetta (41) to model 10,000 structures of both WT and neoepitope peptide–MHC complexes for FAM171b, COX6a2, and SH3RF1 from template structures PDB 1G7P (42), 2VAB (43), and 4PGE (44), respectively. Principal component analysis (PCA) was conducted on peptide-only cartesian coordinates of all 10,000 decoys for each peptide modeled, and principal components 1–3 were clustered with the density-based spatial clustering of applications with noise (DBSCAN) algorithm (45) using ε of 1.5 and a minimum cluster size of 40. From the most populous non-noise cluster, the model with the lowest ref2015 score was retained as a representative model for subsequent evaluation and comparison. Root-mean-square deviations (RMSDs) of atomic positions of peptide common or backbone heavy atoms between WT and mutant peptides were calculated, and models were inspected visually for differences in structural features with PyMOL or Discovery Studio. Peptide SASA and hydrophobic SASA (hSASA) in the context of the MHC I were calculated in Rosetta with a probe radius of 1.4 Å.

Molecular dynamics simulations were performed as described previously (46). Briefly, simulations were performed with GPU-accelerated AMBER 18 (47) and the ff14SB force field (48), with the final models for each peptide/MHC from Rosetta used as starting coordinates. Systems were brought to an NaCl concentration of 0.150 M and solvated in explicit SPC/E water (49) with box edges a minimum of 15 Å from protein atoms. A 12 Å cutoff was used for nonbonded interactions. These were brought to local energy minima, heated to 300 K under restraints, then equilibrated in an isothermal-isobaric (NPT) ensemble with stepwise relaxation of restraints. After a final equilibration in a constant-temperature, constant-volume (NVT) ensemble, production simulations were conducted in an NVT ensemble for 300 ns. RMSDs and root-mean-square fluctuations (RMSFs) of atomic coordinates, as well as ensemble-average structures, were calculated with the cpptraj utility in AmberTools. Electrostatic surface potentials were calculated using pdb2pqr (50) and the APBS software suite (51) with grid spacing of 0.25 Å at a temperature of 310 K and salt concentration of 0.150 M.

### Isolation of MHC-presented peptides from cells for MS.

MHC I–β2-microglobulin-peptide complexes were isolated from 1 × 10^9^ BMDCs pulsed with the 100 μM 21-mer peptides, as described previously (52). After 1 hour of incubation, cells were washed with ice-cold PBS and pellets frozen at −20°C. The frozen pellet was resuspended in ice-cold lysis buffer (20 mM Tris HCl, 150 mM NaCl, 1% Triton X-100, 0.1% octyl glucoside, and protease inhibitor cocktail) and incubated for 30 minutes at 4°C. Lysate was centrifuged at 12,000*g* for 20 minutes at 4°C, and loaded onto a protein G–Sepharose column (without bound antibodies) to remove any existing immunoglobulins. The cleared lysate was immediately loaded into the prepared protein G–Sepharose with covalently bound anti-MHC antibody. This column was incubated for 1 hour at 4°C. The column was washed with 10 mL of buffer A (20 mM Tris HCl, 150 mM NaCl) followed by 10 mL of buffer B (20 mM Tris HCl, 400 mM NaCl), then 10 mL of buffer A again, and lastly 10 mL of buffer C (20 mM Tris HCl). Bound MHC I–β_2_-microglobulin-peptide complexes were eluted in 0.5 mL fractions using 0.1N acetic acid.

Eluted proteins were separated from peptides on a Sep-Pak cartridge. The cartridge was washed with 80% acetonitrile in 0.1% trifluoroacetic acid (TFA) and 2 additional times with 0.1% TFA. The eluates were applied and the column was washed with 0.1 TFA. Peptides were eluted in 30% acetonitrile in 0.1% TFA; MHC I and β2-microglobulin were eluted subsequently in 80% acetonitrile in 0.1 % TFA. The peptides were vacuum dried at 37°C and stored at –20°C.

### MHC-bound peptide analysis using ultra-high-performance liquid chromatography and high-resolution tandem MS.

Dried, desalted peptides were resuspended in 0.1% formic acid in water and analyzed using nanoflow ultra-high-performance liquid chromatography coupled to tandem MS (MS/MS). One microliter of desalted peptides was loaded on a 75 μm × 25 cm Easy Spray PepMap C18 analytical column (Thermo Fisher Scientific) held at 35°C and subjected to a 1 hour, 300 nL/min flow linear gradient. Gradient conditions were as follows: 4% solvent B hold for 10 minutes, ramp to 30% solvent B in 40 minutes, 30% solvent B to 90% solvent B in 10 minutes (solvent A: 0.1% formic acid in water, solvent B: 0.1% formic acid in acetonitrile) on a Dionex Ultimate RSLCnano UPLC system. Eluted peptides were directly ionized into a Q Exactive HF hybrid mass spectrometer (Thermo Fisher Scientific) using electrospray ionization and a +1.9 kV spray voltage.

The Q Exactive HF was operated in positive mode and we implemented a data-dependent acquisition method composed of a single full MS scan followed by 15 MS/MS scans. Full MS scans used the following parameters: mass range 300 to 1800 *m*/*z*, 60,000 resolution, default charge state 2, 1 microscan, and 1 × 10^6^ AGC target. Data-dependent MS/MS scans used the following parameters: 1 microscan, 15,000 resolution, 1 × 10^5^ AGC target, maximum IT of 40 ms, 2.0 *m*/*z* isolation window, 0.0 *m*/*z* isolation offset, normalized collision energy of 27, and dynamic exclusion set to 30 seconds.

### Bioinformatic identification of peptide sequences analyzed using Byonic.

Byonic v3.1 (Protein Metrics Inc.) was used to search the raw MS data against a custom proteome database composed of the Uniprot *Mus* *musculus* proteome (UP000000589, accessed May 16, 2017) and manually added peptide sequences of the 21-mer TRMN-containing peptides that were pulsed onto BMDCs. The common proteomics contaminants Byonic database and decoy database were also searched. The following parameters were used: nonspecific enzyme specificity, 5 ppm precursor and 20 ppm fragment mass tolerances, oxidized Met and N-terminal acetyl variable modifications, 2,000 Da maximum precursor mass, compute precursor and charge assignments from MS1, automatic score cut (5% peptide spectrum match [PSM] FDR cuts) enabled, and no protein level FDR cuts. All other parameters were kept at default values. The Byonic-reported peptide hits were manually exported from Byonic Viewer and sorted by FDR 1D to identify pulsed peptide sequences ranked below 5% PSM FDR. The peptide hit for pulsed BMDC sequence EVSGVHRFF exceeded the PSM FDR cutoff (score 147.6, 0.015 FDR 1D, 0.0085 FDR 2D) and was subject to visual inspection. To increase confidence of the identification, the MS/MS spectrum matched to EVSGVHRFF was then compared with that for a synthetic peptide with identical sequence using the UPLC-MS/MS methods described above.

### Isolation of TILs.

Tumors were harvested and dissociated using the Miltenyi Biotec tumor dissociation kit for mouse. CD8^+^ TILs were isolated with STEMCELL’s EasySep murine CD8 negative selection kit.

### scRNA-Seq library generation.

Single cells were then captured for subsequent scRNA-Seq and library preparation as follows: 12,000 single cells were loaded for capture using a Chromium Single Cell 5′ v1.0 (10× Genomics). After capture and lysis, cDNA was synthesized and amplified (12 cycles) as per the manufacturer’s protocol. The amplified cDNA was then divided and used to construct 3 gene expression libraries and 3 V(D)J T cell–enriched libraries as per the manufacturer’s protocol. All libraries were sequenced on a NextSeq 550 system (Illumina) following 10× Genomics’s suggested read length and depth. The Cell Ranger Single-Cell Software Suite v.3 (10× Genomics) was used to perform sample demultiplexing, barcode processing, and single-cell 5′ counting.

### scRNA-Seq alignment, barcode assignment, and UMI counting.

Cell Ranger v.3 count pipeline was used to process the FASTQ files for each sample. The mm10 genome and transcriptome were used to align samples, filter, and quantify. The cellranger aggr pipeline was used to aggregate the analysis files for each sample into a combined set by performing between-sample normalization (samples were subsampled for an equal number of confidently mapped reads per cell). Cell Ranger pipeline output, the “feature (gene) vs. cell” count matrix, was then used for the secondary scRNA-Seq analysis in SC1 as described below.

### Preprocessing analysis.

After the SC1 pipeline for scRNA-Seq analysis (available at https://sc1.engr.uconn.edu/), secondary quality control (QC) was applied to the combined dataset of balanced number of cells per library (constructed by randomly sampling approximately 5000 cells from each library before QC). Genes that were expressed in fewer than 10 cells were excluded from the analysis, and to reduce outliers, cells that expressed fewer than 500 and more than 6000 genes were excluded. Other QC metrics included examining the fraction of reads mapping to mitochondrial genes; cells were excluded if more than 30% of their unique molecular identifier (UMI) counts were from mitochondrial genes, and cells with less than 5% of counts in ribosomal protein genes were also excluded. Filtered and log-transformed (log_2_[*x* + 1]) count matrix was used in PCA and the first 50 principal components were used as input for t-SNE dimensionality reduction algorithm to obtain a 3D representation of the cells used for the 3D visualization plots.

### Clustering and cluster annotation.

Most informative genes for clustering were picked by their high average TF-IDF scores as described previously (53); hierarchical clustering algorithm using Ward linkage and top average TF-IDF scoring genes as features were used to identify 8 clusters. The top average TF-IDF genes were also used as features for PCA analysis followed by t-SNE projection analysis for the 3D t-SNE data set representation. To characterize clusters, differential expression analysis was done by one-versus-the-rest *t* tests (with Welch/Satterthwaite approximation and 0.95 confidence interval) for each cluster using the *t* test available in R stats package. We also compared the differentially expressed genes for effector clusters (C3, C5, and C8) versus C2 and effector/exhausted clusters (C1, C4, and C6) versus C2, for which the full list of differentially expressed genes is provided in Supplemental Data 3 and 4. All differential expression analyses used log_2_ fold change cutoff of 2 and a *P* value cutoff of 0.01. Functional enrichment analysis was performed using the gProfileR R package to inform cluster annotation. Cluster stability was evaluated using the Dunn index, a metric for evaluating clustering algorithms aiming to evaluate compactness of the clustering. The Dunn index showed a value of 0.6297628 (Supplemental Table 2). We also evaluated the cluster separation matrix (Supplemental Figure 4), which includes the separation values between all pairs of clusters, where the separation is defined as the vector of cluster-wise minimum distances of a point in the cluster to a point of another cluster. This analysis showed that clusters were well-separated from one another.

### TCR sequencing analysis.

Specificity groups/clusters in the TCR repertoire were identified via computational analysis following the GLIPH algorithm from Glanville et al., which searches for global and local motif CDR3 similarity in TCR CDR regions with high contact probability (54). Each specificity group was analyzed in GLIPH for enrichment of common V-genes, CDR3 lengths, clonal expansions, motif significance, and cluster size. Global similarity measured CDR3 differing by up to 1 amino acid and local similarity measured the shared enriched CDR3 amino acid motifs with more than 10-fold enrichment and probability less than 0.001. More details about the algorithm can be found in Glanville et al. (54). Supplemental Data 2 shows the enriched CDR3 motifs of TCRs from CD8^+^ TILs isolated from BMDC-, FAM171b-, and Cd9-immunized mice.

### Clustering analysis of mutant and WT IC_50_ values.

For normalization, simple centering and scaling was performed for the WT and mutant IC_50_ sets. Centering was done by subtracting the column means, and then scaling was done by dividing the (centered) values by their standard deviations. Using the scaled and centered WT and mutant IC_50_ values of all tested FABF peptides and peptides from published works as features, 3 clusters/ellipses were fitted using model-based clustering based on parameterized finite Gaussian mixture models from the mclust (55) package in R; the number of clusters was determined by analyzing the Bayesian information criterion (BIC). Top models based on the BIC criterion were VVI at 3 clusters with BIC value 21.727154 and VEI, also at 3 clusters with BIC value 7.150494.

### Data availability.

Single-cell data used in this paper are provided as a publicly accessible data set (MC38-FABF, ref. 29) at https://sc1-dev.engr.uconn.edu/ scRNA-Seq data of this paper have been submitted to NCBI’s Gene Expression Omnibus (GEO GSE162432).

### Code availability.

The SC1 tool used for scRNA-Seq analysis in this paper is publicly available at https://sc1.engr.uconn.edu/ Custom code for the TCR analysis and cluster analysis of TRMNs (Figure 4) is available upon request and deposited on GitHub at https://github.com/marmarmoussa/FABF

### Statistics.

*P* values for group comparisons were calculated using a 2-tailed nonparametric Mann-Whitney test using GraphPad Prism 5.0. Fisher’s exact test was used to test association between pairs of categorical parameters. Statistical significance of a Pearson correlation coefficient was computed using 2-tailed Student’s *t* test as described in Cohen et al. (56). Statistical significance of a Pearson correlation coefficient was computed using 2-way ANOVA with Sidak’s multiple-comparison test. Statistical analysis of percentage survival curves was conducted using the log-rank (Mantel-Cox) test. For figures where *n* is less than 10 and individual points are not shown, please refer to raw data file in the supplement. *P* values of less than 0.05 were considered significant. Tukey’s multiple-comparison test was done when multiple comparisons were made.

### Study approval.

Mice were maintained in the virus-free mouse facilities at the University of Connecticut Health Center and their use was approved and monitored by the IACUC.

## Author contributions

CAB and MMG contributed equally to this paper. CAB was assigned as the first among co–first authors because he joined the study before MMG. Conversely, MMG continued the study longer. All authors reviewed the manuscript. PKS, IIM, CAB, and MMG conceptualized the initial plan of study, designed the research, analyzed the data, and wrote the manuscript. MMM and IIM guided the development of the algorithm for prediction of TRMNs on the basis of the experimental data. AS guided the studies on the plasticity and stemness of TILs. BMB guided and GLJK performed the modeling of peptide–MHC I complexes and interpreted the data. ATH created the initial list of all SNVs, tested them by Sanger sequencing, and prepared the peptides. MMM, SAS, and TVS analyzed various sequencing data. JLB guided the mass spectrometry experiments and interpreted the data. RPE performed some tumor rejection experiments.

## Supplementary Material

Supplemental data

## Version 1 12/15/2020

In-Press Preview

## Version 2 02/01/2021

Print issue publication
