# Supplementary material for: An unbiased approach to defining bona fide cancer neoepitopes that elicit immune-mediated cancer rejection
Source: J Clin Invest. 2021 Feb 1;131(3):e142823. doi: 10.1172/JCI142823 (PMC7843235; doi:10.1172/JCI142823)
Supplement: Supplemental data [file jci-131-142823-s111.pdf]

This PDF file includes:

Supplementary Figures S1 to S6
Table S1 to S2
Data S1 to S4

Fig. S1.

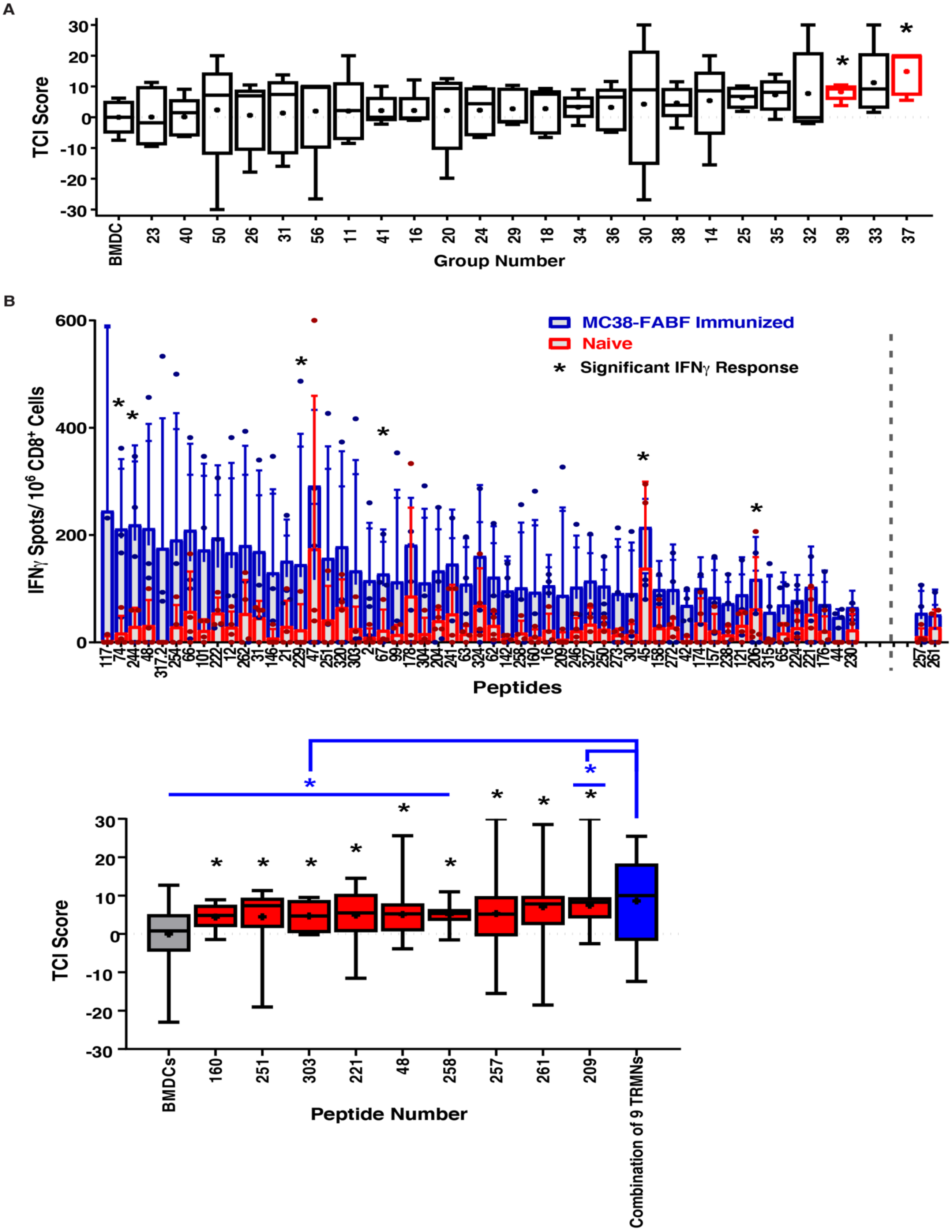

A
TCI Score
30
20
10
0
-10
-20
-30
BMDC
23
40
50
26
31
56
11
41
16
20
24
29
18
34
36
30
38
14
25
35
32
39
33
37
Group Number
B
600
400
200
0
IFNγ Spots/ 10^6 CD8^+ Cells
MC38-FABF Immunized
Naive
* Significant IFNγ Response
117
74
244
48
317.2
254
66
101
222
12
262
31
146
21
229
47
251
320
303
2
67
99
178
304
204
241
63
324
62
142
258
160
16
209
246
327
250
273
30
45
158
272
42
174
157
238
121
206
315
65
224
221
176
44
230
257
261
Peptides
C
TCI Score
30
20
10
0
-10
-20
-30
BMDCs
160
251
303
221
48
258
257
261
209
Combination of 9 TRMNs
Peptide Number

Fig. S1 | Unbiased testing and identification of tumor rejection mediating neoepitopes

(A) BMDCs were pulsed separately with 5 different peptides and pooled together. Mice were immunized prophylactically as described. Box and whisker plot representing the tumor control index (TCI) scores for the 24 of all 56 groups (5 peptides per group) tested which showed a positive TCI score. The negative control (extreme left) consists of mice immunized with un-pulsed BMDCs. n= 5 mice per group; data represented as means ± s.d. shown; (red bars) *$p<0.05$ (student's t- test). **(B)** Bar graph representing top 60 $CD8^+$ T cell response eliciting peptides in the MC38-FABF immunized mice. Splenocytes pulsed with each individual peptide were used as targets. Each pair of blue and overlaying red bars represents the response in MC38-FABF immunized and naïve mice respectively (n=4 mice per group). Statistical analysis was conducted for each peptide's response against wells with no target using 2way ANOVA. Means ± s.d. shown. (C) Box and whisker plot representing the tumor control index (TCI) scores for the nine TRMNs individually (red bars) and combination of all nine TRMNs (blue bar). The negative control (extreme left) consists of mice immunized with un-pulsed BMDCs. n= 10-50 mice per group; data represented as means ± s.d. shown; *$p<0.05$ (student's t- test).

Fig. S2.

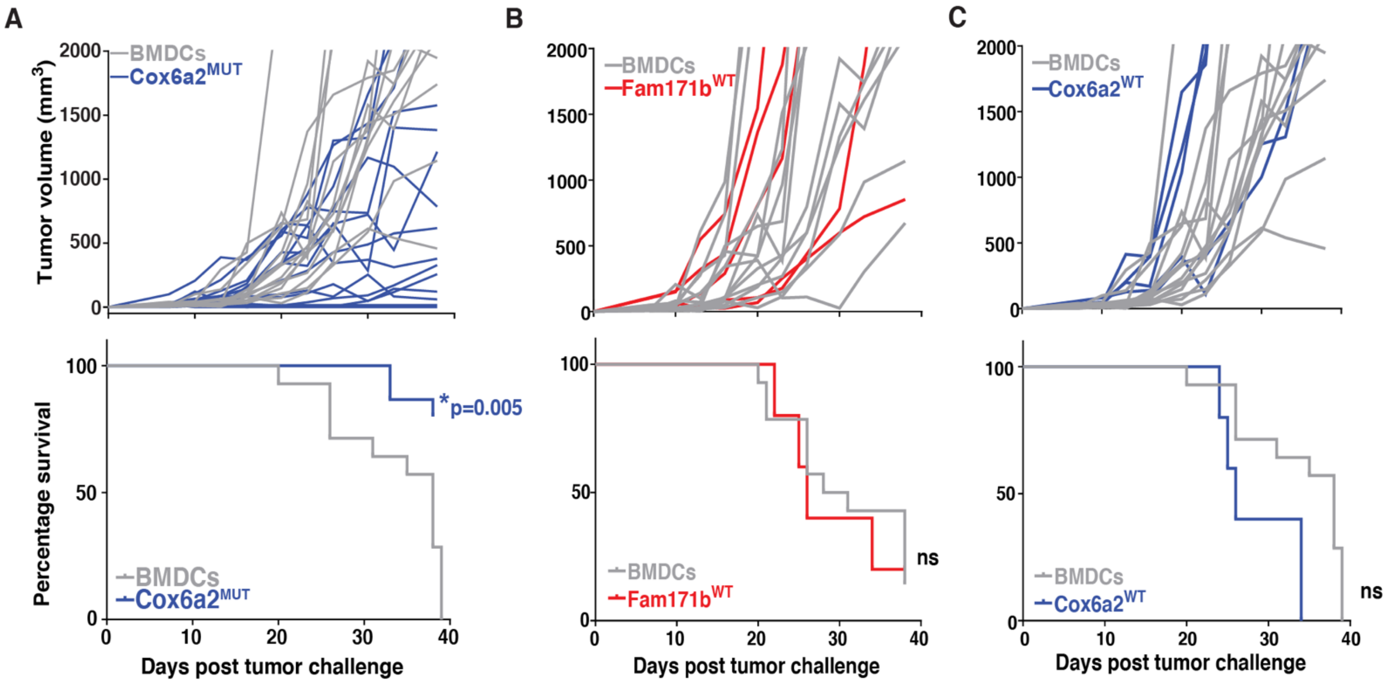

Fig. S2 | Anti-tumor activity of TRMNs derives from the mutations within them. (A) Tumor growth curves (top) and percent survival (bottom) of mice treated on days 10 and 17 post tumor challenge (indicated by arrows) with Cox6a2^{MUT} (blue) or un-pulsed BMDC (grey), n=10 mice / group. Tumor growth curves (top) and percentage survival (bottom) of mice immunized prophylactically with **(B)** wild type Fam171b (red) or **(C)** wild type Cox6a2 (blue) and un-pulsed BMDC controls (grey). Data represented as means ± s.e.m.; n=5-15 mice per group. For survival plots **(A, B and C)**, statistical analysis was preformed using the log rank (Mantel-Cox) test; *$p<0.05$.

Fig. S3.

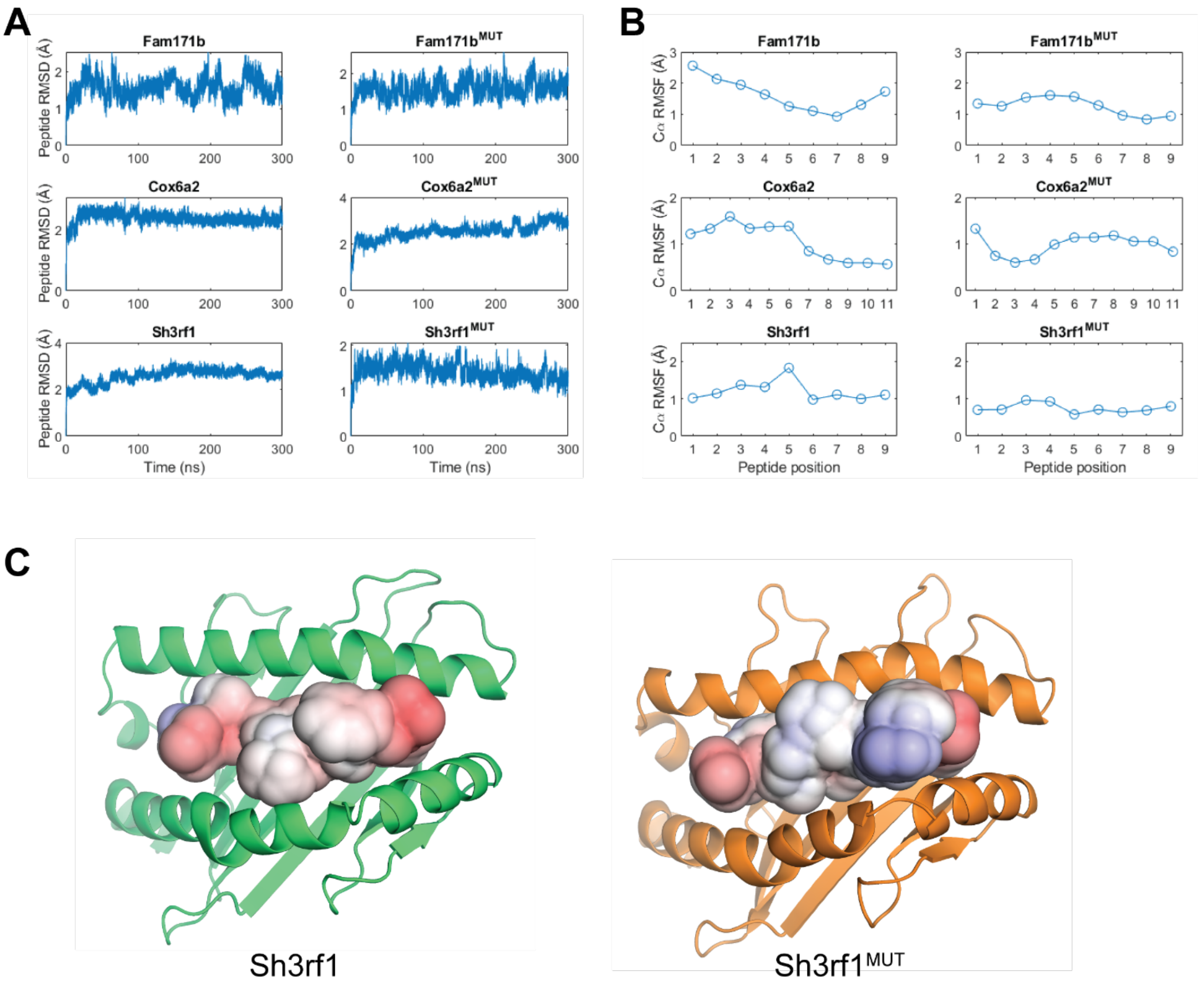

Fig. S3 | Molecular dynamics simulations of the WT and mutant FAM171b, COX6a2, SH3RF1 peptide/K^b complexes.

(A) All atom root mean square deviations (RMSD) and **(B)** Cα root mean square fluctuations (RMSF) from molecular dynamics simulations of the modeled peptide/K^b complexes. RMSD values are calculated for all non-hydrogen peptide atoms after superimposing trajectories on the initial coordinates of the binding groove Cα atoms (residues 1-181) from structural modeling. RMSF was calculated for Cα atoms of the peptide, representing a time-averaged RMSD from the mean position. After deviating approximately 2 Å from the structural models, peptides appear to fluctuate around stable conformations, exhibiting similar properties to those seen in the static models. **(C)** Electrostatic surface potentials of the peptides from the conformation closest to the simulation average for the wild type (left) and mutant (right) SH3RF1 complexes. Surface potentials are on a scale of -4 (blue) to +4 (red) $k_B T/e$. Echoing what is shown in Fig. 3C, the G7R mutation in SH3RF1^{MUT} results in the presentation of a strikingly different surface.

Fig. S4.

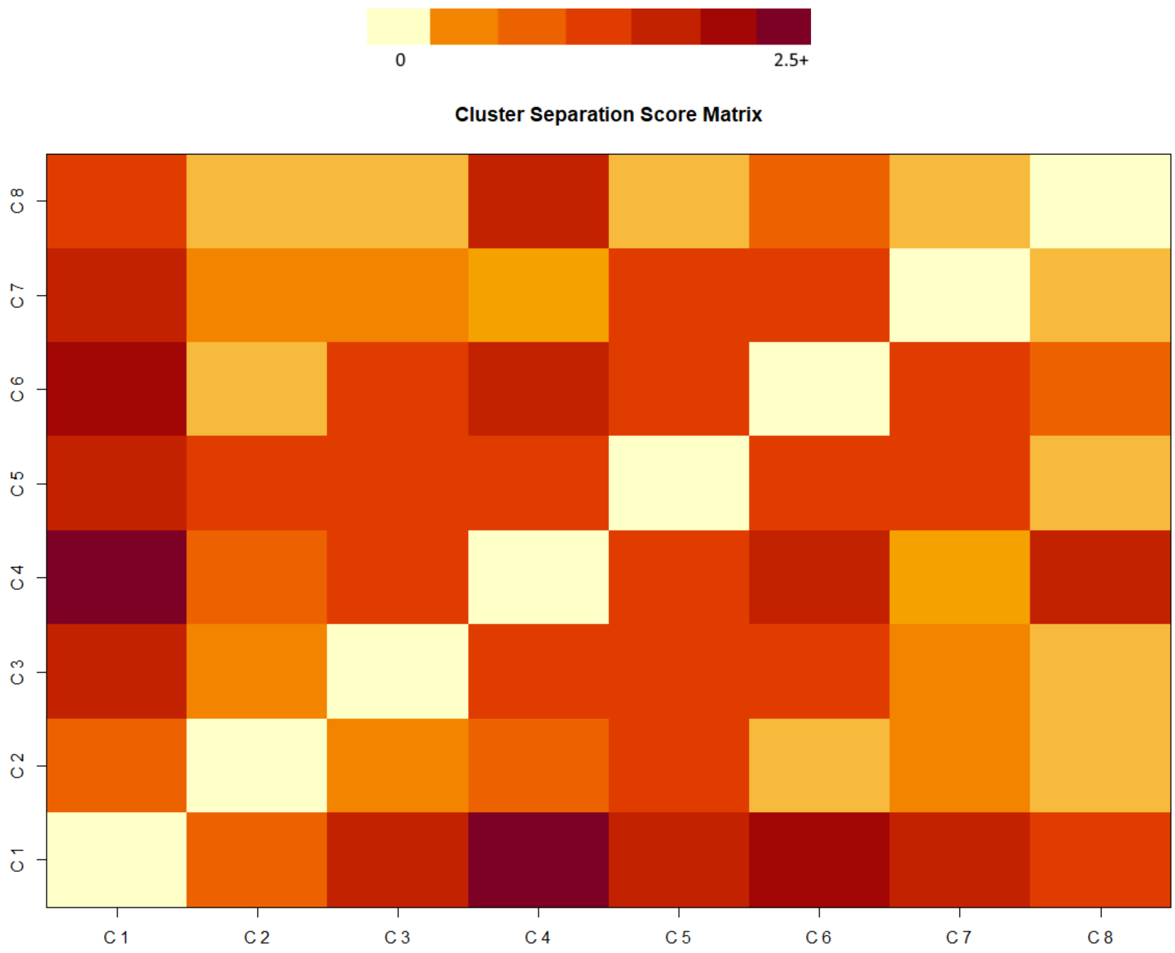

Figure S4 | Cluster separation Matrix analysis for the clusters identified in Figure 5

The matrix includes the separation values between all pairs of clusters, where the separation is defined as the vector of cluster wise minimum distances of a point in the cluster to a point of another cluster.

Figure S5.

A

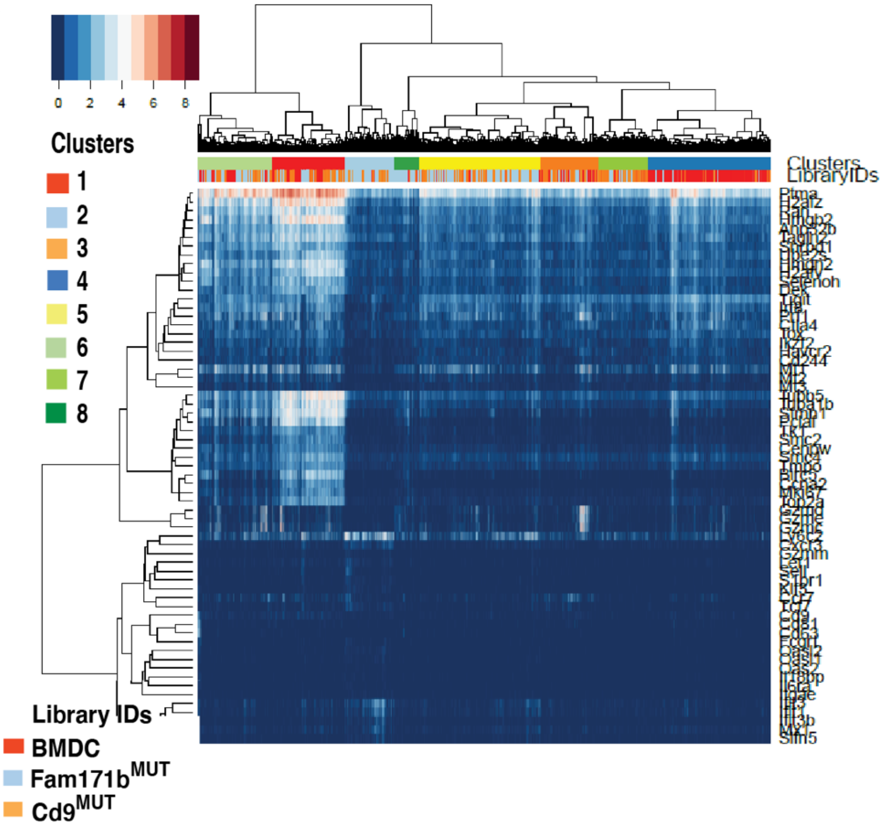

Figure S5 | ScRNA-seq analysis of CD8⁺ PD1⁺ TILs from TRMN and non-TRMN immunized mice.

Mice (n=3 per group) were immunized with un-pulsed BMDCs or BMDCs pulsed with peptides Fam171b^{MUT} (a TRMN) or Cd9^{MUT} (a non-TRMN) and challenged with MC38-FABF. Tumors were harvested day 25 post tumor challenge and live CD8^{+}PD-1^{+} TILs isolated by FACS and sequenced by scRNA-seq (10x genomics). Approximately 4400 CD8^{+} PD-1^{+} TILs were analyzed in each library. **(A)** Heatmap depicting the clustering of CD8^{+}PD-1^{+} TILs (single cells) and differentially expressed (DE) genes.

Fig. S6.

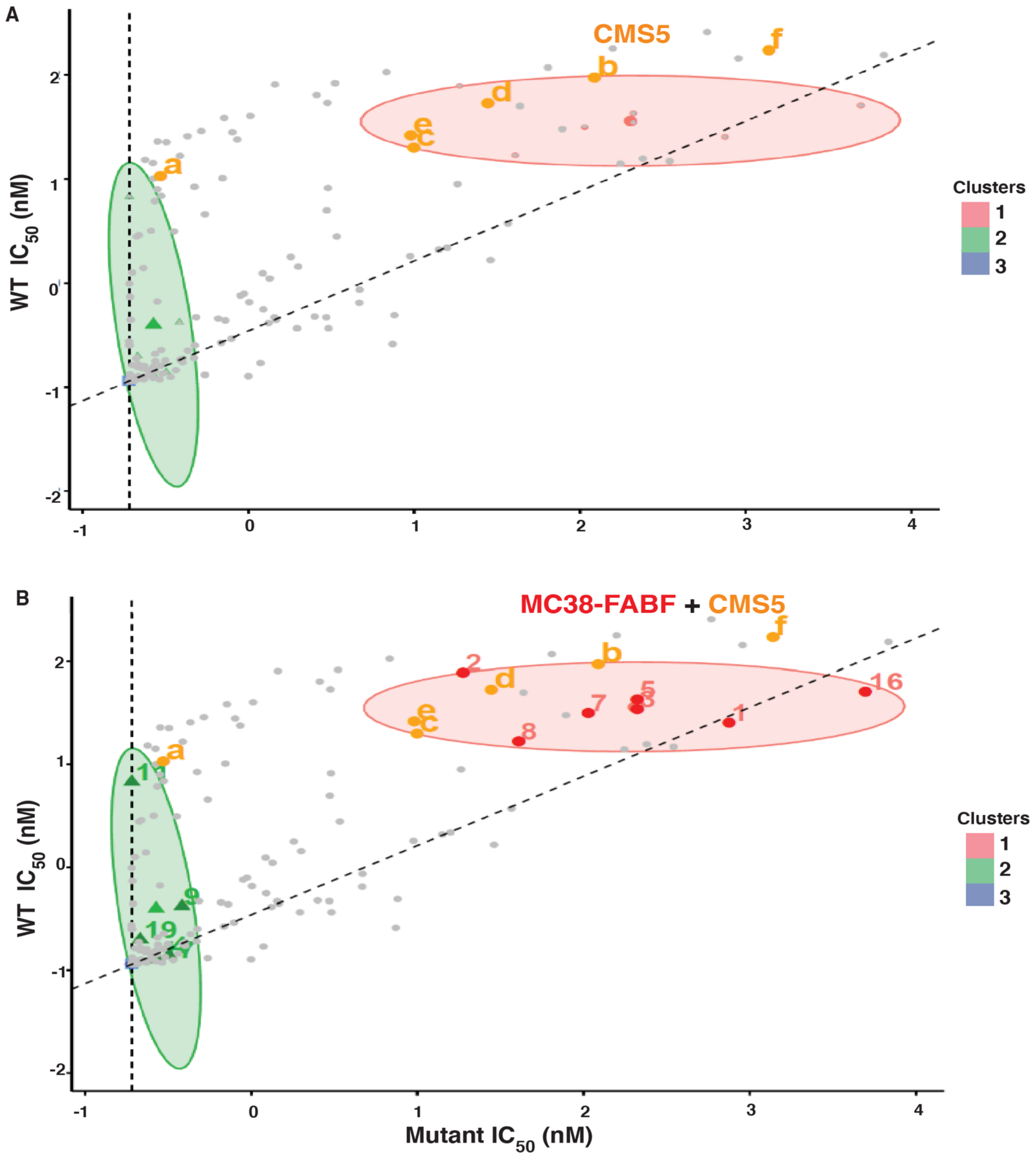
A
CMS5
a
b
c
d
e
f
WT IC50 (nM)
Clusters
1
2
3
B
MC38-FABF + CMS5
1
2
5
7
8
16
19
Mutant IC50 (nM)

Fig. S6 | TRMNs of an independent tumor (BALB/c fibrosarcoma CMS5) map to cluster 1, in a manner identical to the TRMNs of MC38-FABF.

(A) Published data on six TRMNs for the CMS5 sarcoma (Duan et al., 2014) were analyzed and plotted as in Fig. 6C. All TRMNs fall within or close to the boundary of Cluster 1 as in Fig 6C.

(B) TRMNs in Fig 6C and Fig. S4A (above) are plotted together showing that all TRMNs from the two tumors fall within a similar, and mostly identical, boundary.

Table S1 | Interaction of the nine TRMNs with cognate MHC I alleles

Peptide #	Gene name	Predicted IC_{50}[A]		DAI[B]		TPM[C]
		($H\text{-}2K^b$)	($H\text{-}2D^b$)	($H\text{-}2K^b$)	($H\text{-}2D^b$)	
209	Fam171b	17930.3	26004	1.09	0.88	3.72
261	Cox6a2	27341	44317.3	0.29	0.03	18.57
257	Psma1	27412.7	24703.7	0.15	0.41	93.78
258	Plk1	2759.4	18038.9	1.46	1.18	91.60
48	Kif3a	2050.1	25588.9	-0.77	-0.07	14.35
221	Oas3	27345.7	27856.1	0.34	-0.03	16.86
303	Sh3rf1	32309.8	48380	-0.04	-0.01	16.65
251	Tpra1	14.8	9326.3	0.76	0.58	35.45
160	Atg9a	9935.5	45159.9	0.42	0.04	35.01

[A] IC_{50} scores (nM) for $H\text{-}2K^b$ and $H\text{-}2D^b$ are predicted using the NetMHC4.0 algorithm.

[B] DAI scores are calculated as described by Duan *et al* (2014).

[C] Transcripts per million

Table S2| Single Cell Cluster Stability Analysis for clusters in Figure 5

Number of Cells: 13380
Number of Clusters: 8
Cluster sizes (C1 to C8): 1690 1173 1356 2859 2855 1735 1148 564
Dunn Index 0.6297628

Cluster Separation Matrix*:

**C8**	1.73	1	1	2	1.01	1.53	1	0
**C7**	2	1.41	1.41	1.16	1.74	1.73	0	1
**C6**	2.28	1	1.78	2.13	1.83	0	1.73	1.53
**C5**	2.08	1.83	1.75	1.75	0	1.83	1.74	1.01
**C4**	2.59	1.54	1.83	0	1.75	2.13	1.16	2
**C3**	2	1.47	0	1.83	1.75	1.78	1.41	1
**C2**	1.53	0	1.47	1.54	1.83	1	1.41	1
**C1**	0	1.53	2	2.59	2.08	2.28	2	1.73
	**C1**	**C2**	**C3**	**C4**	**C5**	**C6**	**C7**	**C8**

*For cluster stability we evaluated the Dunn Index (Dunn Index for the presented single cell clustering analysis: 0.6297628), a metric for evaluating clustering algorithms aiming to evaluate compactness of the clustering, a value closer to 1 indicate higher between clusters dissimilarity and lower within-cluster dissimilarity, indicating well separated clusters, where the means of different clusters are sufficiently far apart, as compared to the within cluster variance. The Dunn index (calculated from fpc R package) is defined as the minimum average dissimilarity between two cluster divided by maximum average within-cluster dissimilarity. The distance function used is the cosine distance as used for the TF-IDF-based clustering analysis.

Data S1. (Separate file)
Complete list of SNVs called by Epi-Seq.

Data S2. (Separate file)

Table showing the list of CDR3 motifs from TCRs of mice immunized with BMDCs, Fam171b^{MUT} and Cd9^{MUT} libraries.

Data S3. (Separate file)

List DE genes between clusters 1,4 and 6 versus cluster 2

Data S4. (Separate file)

List of DE genes between clusters 3, 5 and 8 versus cluster 2
